# Ultra-Processed Food Consumption and Metabolic Dysfunction-Associated Steatotic Liver Disease (MASLD): Evidence Synthesis, Methodological Appraisal, and Mechanistic Insights

**DOI:** 10.3390/nu18213585

**Published:** 2026-10-29

**Authors:** Melvin Bernardino, Claudio Tiribelli, Natalia Rosso

**Affiliations:** 1 Metabolic Liver Disease Unit, Fondazione Italiana Fegato, 34149 Trieste, Italy; ctliver@fegato.it; 2 Department of Life Science, University of Trieste, 34100 Trieste, Italy; 3 Department of Science and Technology, Philippine Council for Health Research and Development, Taguig 1631, Philippines

**Keywords:** ultra-processed foods, MASLD, steatotic liver disease, umbrella review, systematic reviews, NOVA classification

## Abstract

**Background/Objectives:** Ultra-processed food (UPF) consumption has been associated with metabolic dysfunction-associated steatotic liver disease (MASLD), but differences in exposure definitions and liver outcomes complicate interpretation. This umbrella review synthesized existing evidence, appraised review methodology, examined primary-publication overlap, and explored proposed biological mechanisms. **Methods:** PubMed, Scopus, and Web of Science were searched from inception to May 2026 for systematic reviews and meta-analyses examining UPF consumption and MASLD-related outcomes in adults. The review methodology was assessed using A Measurement Tool to Assess Systematic Reviews 2 (AMSTAR 2). Primary publications were mapped across reviews, distinguishing total NOVA-defined UPF intake from selected-food and dietary-pattern proxies. Publication overlap was quantified using the corrected covered area (CCA). Findings were synthesized narratively. **Results:** Five systematic reviews were included, with AMSTAR 2 confidence classifications of moderate for one, low for one, and critically low for three. Mapping identified 18 distinct primary publications: 11 assessing total NOVA-defined UPF intake and seven assessing proxy exposures. Higher UPF consumption was generally associated with less favorable liver outcomes, with prospective findings proving more consistent than cross-sectional findings. Publication overlap was very high (overall CCA: 34.72%), limiting the independence of review findings. Other limitations included retrospective NOVA classification, heterogeneous liver-outcome ascertainment, and residual confounding. Proposed biological pathways included excess energy intake, visceral adiposity, insulin resistance, hepatic lipogenesis, and altered gut–liver signaling; their contribution to the observed associations remains unconfirmed. **Conclusions:** Existing evidence suggests an association between higher UPF consumption and adverse liver outcomes relevant to MASLD but does not establish causality or a harmful intake threshold. Future research should prioritize repeated dietary assessments, standardized MASLD ascertainment, and interventions evaluating the replacement of UPFs with minimally processed foods.

## 1. Introduction

Metabolic dysfunction-associated steatotic liver disease (MASLD), formerly known as non-alcoholic fatty liver disease (NAFLD), is now the leading cause of liver-related morbidity and mortality around the world [1]. MASLD covers a wide spectrum of disease ranging from simple steatosis to steatohepatitis, different degrees of liver fibrosis, cirrhosis and hepatocellular carcinoma or liver cancer [2]. MASLD has become a major liver and metabolic health problem because it sits at the intersection of hepatic steatosis, obesity, insulin resistance, type 2 diabetes mellitus (T2DM), dyslipidemia, and broader cardiometabolic dysfunction [3]. MASLD affects approximately 38% of the adult population and 7% to 14% of the pediatric population [4]. It affects a large proportion of adults and is increasing, making prevention through modifiable lifestyle factors a public health priority [5].

MASLD is characterized by the accumulation of excess triglycerides in the liver alongside at least one cardiometabolic risk factor, such as obesity, T2DM, dyslipidemia, or hypertension, and in the absence of significant alcohol intake or other secondary causes of liver fat accumulation [3,6]. Nutrition and diet are central to MASLD because hepatic fat accumulation is closely related to energy balance, adiposity, insulin resistance, lipid metabolism, and inflammatory pathways. Traditional nutrition research has often focused on individual nutrients, such as saturated fat, sugar, or refined carbohydrate intake, or on broad dietary patterns, such as the Mediterranean diet (MedDiet), Dietary Approaches to Stop Hypertension (DASH) diet, and Western dietary patterns. In MASLD research, these approaches have helped identify dietary characteristics associated with liver fat and metabolic dysfunction. Recent systematic evidence suggests that MedDiet [2,7,8] and DASH dietary patterns [9,10] may be protective or beneficial for MASLD, whereas Western dietary patterns characterized by greater intake of sweets, red meat, fast food, and other energy-dense foods are positively associated with MASLD [11]. Foods with similar nutrient profiles may differ in their degree of processing, composition, and how they are consumed. This suggests that dietary quality and dietary-pattern composition are relevant, but it also raises a more specific question: whether the degree of food processing contributes to MASLD beyond traditional nutrient-based explanations.

Ultra-processed foods (UPFs) are commonly defined using the NOVA classification as industrial formulations made largely from substances extracted from foods, derived from food constituents, or synthesized often with cosmetic additives, flavor enhancers, emulsifiers, sweeteners, colorants, and sophisticated packaging [12]. UPFs are not simply “processed foods”; they represent a broader food system category that includes many ready-to-eat or ready-to-heat products, such as packaged snacks, sugar-sweetened beverages, industrial bakery products, reconstituted meat products, confectionery, sweetened dairy desserts, many breakfast cereals, and ready meals. These products are often energy-dense, highly palatable, low in fiber, and high in refined carbohydrates, added sugars, unhealthy fats, and sodium [13,14,15]. Interest in UPFs has increased because its exposure has been associated with multiple adverse health outcomes, especially cardiometabolic outcomes, obesity, T2DM, and mortality [16,17]. These outcomes are highly relevant to MASLD because they share metabolic pathways with liver fat accumulation and disease progression. However, the relationship between UPF intake and MASLD cannot be assumed from cardiometabolic evidence alone. Liver outcomes require separate evaluation because hepatic steatosis, steatohepatitis, fibrosis, cirrhosis, and liver cancer differ in their pathophysiology, latency, diagnostic methods, and susceptibility to confounding.

Several mechanisms may explain the relationship between UPF intake and MASLD. A controlled inpatient randomized trial showed that a UPF diet (NOVA 4) increased ad libitum energy intake and weight gain compared with an unprocessed diet (NOVA 1), even when meals were matched for presented calories, macronutrients, sugar, sodium, and fiber [18]. This supports a pathway in which UPFs may promote excess energy intake and weight gain, both of which are important drivers of MASLD. Added sugars, particularly fructose-containing sugars, may also contribute to hepatic fat accumulation through increased de novo lipogenesis (DNL) and impaired fatty acid oxidation [19]. Food additives and UPF-related effects on the gut microbiome, intestinal barrier, and inflammation have also been proposed, although this evidence is still developing and should be interpreted cautiously [20]. 

Emerging liver-specific observational evidence supports the relevance of UPF intake to MASLD. In a large prospective UK Biobank cohort, higher UPF intake was associated with MASLD incidence, defined using the historical nomenclature (NAFLD) and severe liver disease, as well as adverse baseline levels of several liver-related biomarkers [21]. Despite this growing literature, important uncertainties remain. First, the available reviews differ in their inclusion criteria, with some focusing on NOVA-defined UPFs, while others include fast food or broader dietary patterns, and others combine multiple liver outcomes. Second, the primary studies include cross-sectional, case–control, and prospective designs, which differ in their ability to address temporality. Third, MASLD ascertainment varies across studies, including ultrasound, controlled attenuation parameter (CAP), fatty liver indices, hospital records, and diagnostic coding. Fourth, several reviews include overlapping primary studies, which can make the evidence appear larger than it actually is if overlap is not considered. Finally, advanced outcomes such as fibrosis, cirrhosis, and liver cancer require different interpretation from MASLD occurrence because they are less frequent, develop over longer periods, and are influenced by multiple metabolic and non-metabolic factors. For these reasons, a narrative umbrella review is needed. Rather than producing another pooled estimate, an umbrella review can critically compare existing systematic reviews and meta-analyses, assess whether findings are consistent across evidence types, distinguish NOVA-defined UPFs from related exposures such as fast food, evaluate dose–response and prospective evidence, and identify methodological limitations. This approach is particularly appropriate because the current evidence base is heterogeneous and partially overlapping. 

Therefore, the primary objective of this review is to synthesize current evidence from systematic reviews and meta-analyses on the relationship between UPF consumption and MASLD in adults. The secondary objectives are to extract the primary studies included in each systematic review and to: (a) assess consistency across reviews; (b) compare evidence by study design and exposure definition; (c) examine methods of dietary assessment and MASLD ascertainment; (d) identify key methodological limitations; and (e) discuss biological plausibility and mechanism between UPF intake and MASLD risk, based on evidence cited in the included systematic reviews.

## 2. Materials and Methods

### 2.1. Review Design and Protocol 

This study was conducted as an umbrella review with a narrative synthesis of systematic reviews examining UPF intake and MASLD in adult populations. A written protocol was established before study selection began. The systematic review was the unit of inclusion. Relevant primary studies within eligible reviews were subsequently mapped to characterize the underlying evidence and identify overlap. Primary studies included in the eligible systematic reviews were subsequently extracted and examined to identify overlap and assess consistency across reviews; evidence was compared by study design, exposure definition, dietary assessment method, and MASLD ascertainment; and key methodological limitations were identified. No new meta-analysis of primary studies was performed.

### 2.2. Information Sources and Literature Search 

PubMed, Scopus, and Web of Science were searched from database inception to May 2026 to identify systematic reviews with and without meta-analyses examining UPF consumption in relation to MASLD or related liver outcomes. Search terms for UPF (“ultra-processed food”, “Ultraprocessed food”, “UPF”, and “NOVA”) were combined with terms for liver disease (“MASLD”, “MASH”, “NAFLD”, “NASH”, “hepatic steatosis”, and “liver fat”) and study type (“systematic review” and “meta-analysis”). Studies using the earlier terms NAFLD or metabolic dysfunction-associated fatty liver disease (MAFLD) were considered when they reported a relevant liver outcome. 

### 2.3. Eligibility Criteria

Eligibility criteria were established a priori according to the population, exposure, outcome, and review-design domains relevant to the objectives of this umbrella review. Eligible publications were systematic reviews, with or without meta-analysis, which systematically evaluated the association between UPF consumption and MASLD, including studies using the previous terminology of NAFLD or MAFLD. Reviews primarily involving adults (≥18 years) were eligible. The exposure of interest was overall UPF consumption, primarily defined according to Group 4 of the NOVA food classification system or using a food-processing classification conceptually consistent with NOVA. Reviews remained eligible when the overall review addressed UPF consumption but included some primary studies using alternative or proxy definitions of UPFs. Eligible reviews were required to evaluate MASLD either as the primary outcome or as a separately identifiable outcome within a broader assessment of metabolic or liver-related outcomes. Therefore, systematic reviews examining multiple outcomes, such as metabolic syndrome, insulin resistance, liver fibrosis, or liver cancer, were retained when findings specifically related to MASLD could be independently extracted. Reviews addressing these outcomes without separately reported MASLD data were excluded. MASLD could be identified using liver biopsy, imaging techniques, transient elastography or CAP, validated steatosis indices, or clinically established diagnostic criteria. Primary research articles, narrative reviews, scoping reviews without systematic evidence synthesis, umbrella reviews, protocols, editorials, commentaries, letters, conference abstracts, guidelines, animal studies, and in vitro studies were excluded. 

### 2.4. Study Selection

**Figure 1:**
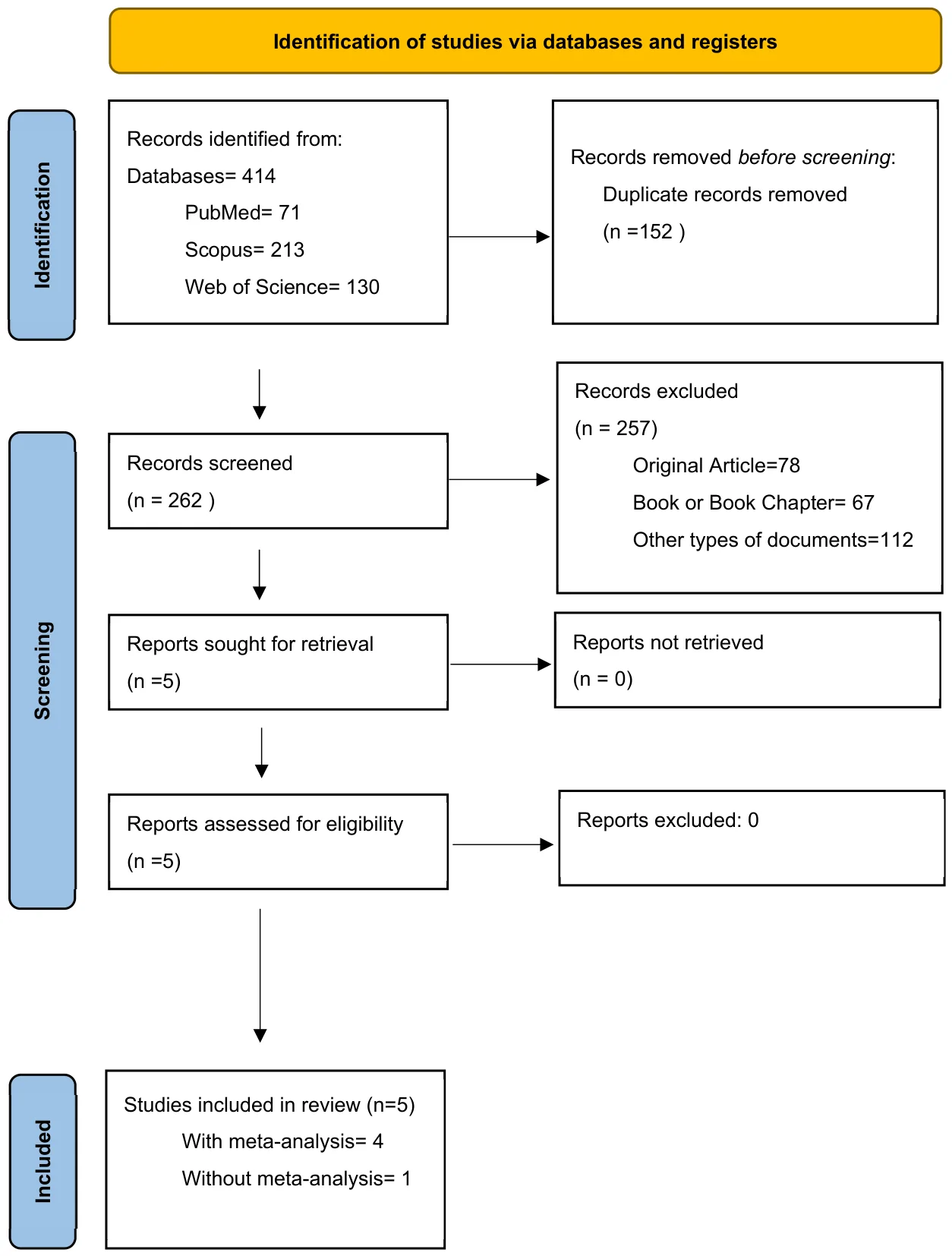
PRISMA Flow Diagram of Screening and Inclusion for the Umbrella Review. This figure has been reproduced from work by Page, McKenzie, Bossuyt, Boutron, Hoffmann, Mulrow, Shamseer, Tetzlaff, Akl, Brennan, Chou, Glanville, Grimshaw, Hróbjartsson, Lalu, Li, Loder, Mayo-Wilson, McDonald, McGuinness, Stewart, Thomas, Tricco, Welch, Whiting, and Moher. This work is licensed under CC BY 4.0. To view a copy of this license, visit: https://creativecommons.org/licenses/by/4.0/ (accessed on 25 October 2026).

Database records were deduplicated using digital object identifiers (DOIs), PubMed identifiers (PMIDs), and title information, with bibliographic checks to distinguish duplicate records from separate reports with similar titles. Two reviewers independently screened titles and abstracts against the eligibility criteria. The same independent assessment procedure was applied to full-text reports considered potentially eligible. Each reviewer made an initial eligibility decision before comparing their decisions with the other reviewer. The first author subsequently verified the extracted information against the original reports. Disagreements were resolved through discussion and re-examination of the eligibility criteria and the relevant reports with the first author. The study selection process is presented in a PRISMA 2020 flow diagram (Figure 1).

### 2.5. Data Extraction and Identification of Primary-Study Overlap

For each included systematic review, data were manually extracted on publication and search dates, the number and design of relevant primary studies, population characteristics, dietary and liver-outcome assessment methods, synthesis methods, principal findings, and stated limitations. Reported pooled estimates, confidence intervals, and heterogeneity statistics were retained. The primary papers contributing to relevant liver outcomes were identified from the reviews’ study tables, results, and reference lists. Their inclusion across the five reviews was recorded in a review-by-study matrix. Studies exclusively addressing other liver diseases were excluded from this matrix. Repeated appearances of the same paper across reviews were counted as overlap, not as an independent confirmation of an association. 

Primary-publication overlap was quantified using the corrected covered area (CCA), calculated as [(N − r)/(rc − r)] × 100, where N represents the total number of primary-publication appearances across reviews, r the number of distinct primary publications, and c the number of reviews [22]. CCA was calculated for the complete inclusion matrix and separately for strictly NOVA-defined UPF exposures and proxy exposures. All five reviews were retained in the principal calculations. Because only two reviews contributed proxy-exposure studies, an additional calculation was restricted to those two reviews. The unit of analysis was the primary publication; overlap in participants across different publications was considered separately.

### 2.6. Methodological Appraisal

The methodological quality of the included systematic reviews was assessed using A Measurement Tool to Assess Systematic Reviews 2 (AMSTAR 2), a 16-item critical appraisal tool developed for reviews of randomized or non-randomized studies of healthcare interventions [23]. Its selection reflected the relevance of its core methodological domains to reviews of dietary exposures and health outcomes and its previous application in nutritional umbrella reviews incorporating observational evidence [24]. Two reviewers independently completed the AMSTAR 2 assessments. Initial item-level ratings and overall confidence classifications were recorded separately before the reviewers compared their assessments. Disagreements were resolved through discussion, with reference to the original AMSTAR 2 criteria and the methods. The first author subsequently verified the extracted information against the original reports. Items were rated as “Yes,” “Partial Yes,” or “No” according to the original criteria, with “Partial Yes” restricted to items permitting this response. “Partial Yes” indicated that the review met the item-specific minimum requirements but not all the requirements for a “Yes” judgment. This response was used only for items permitting partial adherence under the original AMSTAR 2 criteria. For items conditional on meta-analysis, “No meta-analysis conducted” was recorded where appropriate. Overall confidence in review results was classified as high (no critical flaws and no more than one non-critical weakness), moderate (multiple non-critical weaknesses without critical flaws), low (one critical flaw), or critically low (multiple critical flaws), rather than calculated from a numerical score. Multiple non-critical weaknesses were also considered as grounds for downgrading moderate confidence to low.

### 2.7. Narrative Synthesis

Evidence was synthesized at two levels. First, systematic reviews were compared by eligibility criteria, exposure definition, study design, liver outcome, pooled or narrative findings, and methodological quality. Second, their relevant primary papers were mapped to assess overlap and characterize the underlying evidence. Primary studies were synthesized separately according to exposure definition, distinguishing total NOVA-defined UPF intake from selected-food and dietary-pattern proxies. For NOVA-defined studies, the synthesis considered study characteristics, dietary assessment and validation, exposure metrics, covariate adjustment or experimental comparisons, liver-outcome definitions, findings, strengths, and limitations. Outcomes were grouped by ascertainment method, distinguishing imaging-derived liver fat, surrogate steatosis indices, and record-based diagnoses. Correspondence with current MASLD criteria was assessed qualitatively using reported steatosis, cardiometabolic, and alcohol-related criteria, without participant-level reclassification. Proxy-exposure studies were examined separately using the same exposure and outcome distinctions. Recurring methodological limitations and corresponding research priorities were then compared across reviews. 

Proposed mechanisms linking UPF consumption with MASLD were discussed narratively using the included reviews and the mechanistic literature cited in the eligible systematic reviews. Mechanistic pathways were presented as hypotheses; they were not assumed to establish causal mediation of the observed associations. The findings were synthesized qualitatively, highlighting areas of emerging evidence as well as gaps in the existing literature. Reported estimates were retained with their study-specific adjustment sets and interpreted as adjusted associations rather than estimates of a common total or direct causal effect. No additional quantitative synthesis or systematic comparison of sequential adjustment models was undertaken; differences in adjustment were considered when interpreting the evidence. Discrepancies arising during study selection, data extraction, or quality appraisal were addressed through deliberation among the review team and by referring to the underlying evidence. The term MASLD is used in accordance with the updated nomenclature [3,25,26]. The term MASLD is used throughout the results and discussion section, whereas the original outcome terminology and diagnostic definitions are retained when describing individual studies. 

## 3. Results

### 3.1. Methodological Quality of the Included Reviews

AMSTAR 2 ratings varied across the five reviews. Grinshpan et al. (2024) [27] was rated as having moderate confidence, Henney et al. (2023) [28] as having low confidence, and Guo et al. (2025) [29], Zhang et al. (2025) [30], Souza et al. (2026) [31] as having critically low confidence (Supplementary Table S1). All five reviews performed study selection in duplicate and addressed heterogeneity. All four meta-analyses were rated as using appropriate statistical methods. Recurring weaknesses included incomplete search strategies, limited reporting of review protocols, and failure to report the funding sources of included primary studies. Three reviews did not provide a justified list of excluded full-text studies. These methodological limitations reduce confidence in the review-level evidence, even though the reported associations generally point in the same direction.

### 3.2. Ultra-Processed Food Consumption and MASLD: Evidence from Systematic Reviews

Five systematic reviews evaluated the association between UPF consumption and MASLD (Table 1). Four conducted meta-analyses, while Grinshpan et al. [27] used narrative synthesis. Their findings generally pointed toward a positive association, although the reviews differed in whether they measured total NOVA-defined UPF intake or used selected foods and dietary patterns as proxies. Henney et al. (2023) [28] reported a higher risk in the highest versus lowest intake categories [relative risk (RR) 1.42, 95% confidence interval (CI) 1.16–1.75], as did Guo et al. (2025) [29] [odds ratio (OR) 1.72, 95% CI 1.36–2.17]. These estimates require cautious interpretation because both reviews included studies that assessed selected UPF-related foods rather than total UPF intake. In a synthesis restricted to NOVA-defined total UPF, Zhang, Shu and Chen (2025) [30] found a more modest association for the highest versus lowest intake (RR 1.22, 95% CI 1.14–1.31) and for each 10% increment in intake (RR 1.06, 95% CI 1.04–1.09). However, their separate formal dose–response model was not statistically significant. Prospective evidence also supported an association.

Souza et al. (2026) [31] pooled three cohorts and found that higher UPF intake preceded a greater incidence of MASLD [Hazard Ratio (HR) 1.32, 95% CI 1.11–1.58], highest versus lowest intake). Grinshpan et al. (2024) [27] reported positive findings in both large prospective studies included in their narrative review, although only three of its six liver-related papers found statistically significant associations overall. Taken together, the systematic reviews consistently suggest that greater UPF consumption is associated with MASLD. The pooled studies overlap across reviews, so their estimates should not be treated as independent confirmation. Substantial heterogeneity, differences in exposure and outcome definitions, and the predominance of observational evidence limit conclusions about causality or a specific intake threshold.

### 3.3. Primary Research Papers Included in Each Systematic Review

The included reviews generally reported a positive association between UPF consumption and MASLD, but they differed in how UPF exposure was defined. To examine this variation, we mapped the primary papers contributing to MASLD or liver-fat outcomes in each review (Table 2). The five reviews included 18 distinct primary papers, with considerable overlap: Zhang S. et al. (2022) appeared in all five, while Ivancovsky-Wajcman et al. (2021) and Konieczna et al. (2022) each appeared in four. We then distinguished studies measuring total NOVA-defined UPF intake from those assessing selected foods or dietary patterns as proxies. This distinction clarifies which findings directly address the association between overall UPF consumption and MASLD.

The corrected inclusion matrix contained 18 distinct primary publications contributing 43 appearances across five reviews, yielding an overall CCA of 34.72%. The 11 publications assessing strictly NOVA-defined UPF exposure contributed 31 appearances, corresponding to a CCA of 45.45%. The seven proxy-exposure publications contributed 12 appearances, yielding a CCA of 17.86% across all five reviews. When the proxy-exposure analysis was restricted to Henney et al. and Guo et al., the only reviews contributing these publications, CCA increased to 71.43%. These findings indicate very high publication overlap and demonstrate that the reviews draw substantially on shared primary evidence (Table 3).

### 3.4. Assessing Total UPF Intake Using the NOVA Classification

Eleven (11) studies assessed total UPF intake using the NOVA classification (Table 4). Four (4) were cross-sectional, five (5) were prospective cohort studies, (1) one controlled feeding trial, and one (1) was a longitudinal analysis within a randomized lifestyle trial. The studies were conducted across Asia, Europe, North America, and South America. Two analyses used UK Biobank data, and two used overlapping National Health and Nutrition Examination Survey (NHANES) survey periods. 

#### 3.4.1. Dietary Assessment and Validation 

Across the primary studies, UPF intake was measured and reported in several ways. UPF exposure was reported using different measures, including grams per day, intake adjusted for energy, percentage of total energy, and percentage of food weight. Dietary intake was assessed using food-frequency questionnaires (FFQs) in six studies and 24-h food recalls in four studies (Table 4). Ivancovsky-Wajcman et al. (2021) [32] used a 117-item FFQ; Zhang S. et al. (2022) [33] used a questionnaire expanded from 81 to 100 items; Fridén et al. (2022) [34] used a 140-item FFQ; Konieczna et al. (2022) [35] used a 143-item FFQ administered at baseline, 6 months, and 12 months; and Fu et al. (2025) [40] used a 106-item FFQ. Canhada et al. (2024) [39] also used a validated FFQ to assess baseline intake. Among the recall-based studies, Liu et al. (2023) [36] and Zhao, Martinez Steele et al. (2023) [37] used NHANES 24-h dietary recalls, whereas Zhang Y.-F. et al. (2024) [38] and Zhao Clay-Gilmour et al. (2024) [21] used repeated web-based recalls from UK Biobank. 

Several dietary tools used in the ten observational studies had been validated for general dietary assessment, but none reported validation specifically for identifying food-processing level. NOVA categories were assigned retrospectively to FFQ items or recalled foods, creating potential for misclassification when preparation methods or ingredients were insufficiently described. In contrast, Hall et al. (2019) [18] provided NOVA-defined ultra-processed and unprocessed menus in an inpatient crossover feeding trial and measured intake by weighing foods offered and leftovers, without relying on a self-reported dietary instrument.

#### 3.4.2. MASLD Ascertainment

The studies also differed in how they identified liver outcomes (Table 5). Ivancovsky-Wajcman et al. (2021) [32], and Zhang S. et al. (2022) [33] used ultrasonography, whereas Hall et al. (2019) [18] used proton magnetic resonance spectroscopy (MRS), Fridén et al. (2022) [34] measured liver fat by magnetic resonance imaging (MRI). Zhao, Martinez Steele et al. (2023) [37] used transient elastography. Other studies relied on surrogate indices: Konieczna et al. (2022) [35] examined changes in the Fatty Liver Index (FLI) and Hepatic Steatosis Index (HSI), Liu et al. (2023) [36] used the United States FLI, and Fu et al. (2025) [40] defined incident probable NAFLD using an FLI threshold. The two UK Biobank studies identified liver outcomes through linked hospital and death records, capturing clinically recorded disease rather than systematically screening all participants for steatosis. 

Canhada et al. (2024) [39] was the only study to explicitly use a “new MASLD definition”, combining FLI-defined steatosis with at least one cardiometabolic risk factor. Its steatosis assessment nevertheless relied on a surrogate index. The remaining studies used historical NAFLD definitions or examined liver-fat measures, and none reported biopsy-confirmed MASLD. These differences are relevant when interpreting results across studies because the outcomes range from estimated liver fat to imaging-detected steatosis and clinically recorded liver disease. Across the eleven studies, no liver outcome was confirmed by biopsy. Their findings therefore refer to outcomes ranging from continuous liver-fat measures and index-defined probable steatosis to imaging-detected or clinically recorded disease, which should be distinguished when comparing associations with UPF intake.

#### 3.4.3. Association Between NOVA-Defined UPF Intake and Liver Outcomes

Among the eleven studies assessing total NOVA-defined UPF intake, five prospective cohorts examined incident liver disease (Table 6). In the Chinese cohort, Zhang S. et al. (2022) [33] found a higher risk of ultrasound-detected NAFLD in the highest versus lowest quartile of UPF intake (HR 1.18, 95% CI 1.07–1.30). Fu et al. (2025) [40] similarly reported a higher risk of FLI-defined probable NAFLD in the Korean Health Examinees (HEXA) cohort, with estimates of 1.35 (95% CI 1.06–1.71) in men and 1.48 (95% CI 1.19–1.86) in women. Two UK Biobank studies used linked hospital and death records but assessed different outcomes: Zhang Y.-F. et al. (2024) [38] reported an association with severe NAFLD (HR 1.26, 95% CI 1.11–1.43), while Zhao, Clay-Gilmour et al. (2024) [21] reported an association with recorded NAFLD (HR 1.43, 95% CI 1.21–1.70). These estimates came from the same underlying cohort and should not be treated as findings from independent populations. In the Brazilian ELSA-Brasil cohort, Canhada et al. (2024) [39] found that each one-standard-deviation increase in UPF intake, equivalent to 271 g/day in the MASLD analysis, was associated with higher incidence of FLI- and cardiometabolic-criteria-defined MASLD (RR 1.12, 95% CI 1.08–1.16). The four cross-sectional studies produced a less uniform pattern. Using National Health and Nutrition Examination Survey (NHANES) 2011–2018 data, Liu et al. (2023) [36] found higher odds of US FLI-defined probable NAFLD in the highest versus lowest quartile of UPF intake measured as a percentage of food and beverage weight (OR 1.83, 95% CI 1.33–2.53). In NHANES 2017–2018, Zhao, Martinez Steele et al. (2023) [37] found higher odds of transient-elastography-defined NAFLD among adults in the highest versus lowest UPF intake category (OR 1.72, 95% CI 1.01–2.93). The NHANES periods overlap, so these analyses should not be regarded as wholly independent samples. By contrast, Ivancovsky-Wajcman et al. (2021) [32] did not find a significant association with ultrasound-defined NAFLD prevalence.

Among participants who already had NAFLD, however, higher UPF intake was associated with a marker of presumed NASH (OR 1.89, 95% CI 1.07–3.38). In Sweden, Fridén et al. (2022) [34] observed an association between UPF intake and MRI-measured liver fat in crude analyses, but it did not persist after full adjustment. Konieczna et al. (2022) [35] examined changes over one year among older Spanish adults with overweight or obesity and metabolic syndrome. Each 10-percentage-point increase in UPF’s share of total food weight was associated with higher FLI (β 1.60 points, 95% CI 1.24–1.96) and HSI (β 0.43 points, 95% CI 0.29–0.57) scores. These findings concern changes in surrogate indices rather than diagnosed incident disease. Overall, the prospective studies provide the most consistent evidence of an association. Interpretation remains limited by retrospective NOVA classification of dietary data, potential residual confounding, and differences between imaging, surrogate-index, and administrative-code liver outcomes.

### 3.5. Studies Included in Reviews but Not Directly Assessing Total NOVA-Defined UPF

Seven observational studies assessed selected foods, meals, or dietary patterns considered relevant to UPF consumption (Table 7). These comprised three case–control studies [43,44,46], two cross-sectional studies [41,47], a nested case–control analysis [42], and an analysis of repeated fast-food-frequency reports in the Coronary Artery Risk Development in Young Adults (CARDIA) cohort [45]. 

The exposures in the observational studies ranged from red or processed meat [41,42,46] and energy-dense snacks [43] to fast-food meals [45] and dietary patterns containing sweets, soft drinks, or processed meat [44,47]. Most studies identified MASLD using ultrasonography, CAP measurements, or administrative claims. Odegaard et al. (2022) [45] instead assessed MASLD at the year-25 examination using CT-measured liver attenuation and metabolic criteria (Table 8). Because the dietary measures did not classify and sum all foods according to NOVA, their associations describe the specified foods, meals, or patterns rather than overall UPF intake. Greater intake of several of these exposures was associated with less favorable liver outcomes. The highest versus lowest intake categories were associated with higher MASLD odds for red and/or processed meat in Zelber-Sagi et al. (2018) [41] (OR 1.47, 95% CI 1.04–2.09), energy-dense snacks in Yari et al. (2020) [43] (OR 2.27, 95% CI 1.19–4.31), and processed red meat in Noureddin et al. (2020) [42] (OR 1.18, 95% CI 1.05–1.32). Tutunchi et al. (2021) [44] associated a pattern rich in sweets, hydrogenated fat, red and processed meat, and soft drinks with higher MASLD odds; Xiao et al. (2023) [47] reported a similar association for a Western dietary pattern. The CARDIA analysis [45], reported that frequent fast-food consumption was associated with MASLD at year 25 (OR 5.18, 95% CI 2.87–9.37 for ≥3 meals per week versus ≤1 per month). Thus, all seven studies are relevant to understanding the evidence assembled by the reviews, but their findings address selected dietary exposures or short-term responses to assigned diets.

### 3.6. Methodological Limitations of the MASLD Evidence Base and Priorities for Future Research 

Across the five systematic reviews, recurring limitations concerned study design, liver-outcome ascertainment, dietary assessment, geographic representation, and reporting of UPF exposure. Given the available data, the study summarizes the principal limitations and corresponding research priorities, which are discussed by methodological domain below (Table 9).

#### 3.6.1. Type of Study Available

The five systematic reviews included predominantly observational evidence. When we extracted the primary studies within the systematic reviews, eleven studies were identified that directly assessed total NOVA-defined UPF intake. Among the eleven studies, four were cross-sectional studies, five were prospective cohorts, one was a controlled feeding trial and one was a longitudinal observational analysis within a randomized lifestyle trial. Prospective studies generally reported a positive association between higher UPF intake and incident liver outcomes, whereas cross-sectional findings were less consistent. The available study designs of the research limit the ability to establish whether UPF intake preceded disease onset and leave the findings vulnerable to reverse causality and residual confounding. Larger prospective cohorts with repeated dietary measurements and incident MASLD outcomes were the most consistent research priority across reviews.

#### 3.6.2. MASLD Diagnosis

Liver outcomes were ascertained using ultrasonography, MRI, transient elastography, fatty liver indices, or hospital and death records. These methods captured different outcomes, from measured liver fat to clinically recognized disease, and should not be treated as diagnostically equivalent. Most studies used the historical NAFLD definition or a related liver-fat outcome. Future studies should apply explicit MASLD diagnostic criteria, report how steatosis and cardiometabolic risk factors were established, and distinguish incident disease from prevalent disease. Where feasible, quantitative imaging should be used to assess liver fat, alongside validated measures of fibrosis and disease severity.

#### 3.6.3. Type of Dietary Assessment and UPF Exposure

Dietary tools should capture food brands, ingredients, preparation methods, and product changes sufficiently to support reproducible, product-level NOVA classification. Investigators should assess total NOVA Group 4 intake separately from selected UPF subgroups or individual foods, and report exposure in comparable units, particularly percentage of total energy intake and grams per 1000 kcal. Repeated dietary assessments would improve estimates of habitual intake. Studies should also examine dose–response relationships and report which minimally processed foods replace UPFs in substitution analyses. These steps would reduce exposure misclassification and make results easier to combine across studies.

#### 3.6.4. Regional Differences

Direct NOVA studies were conducted in Israel, China, Sweden, Spain, the United States, the United Kingdom, Brazil, and South Korea. In Guo et al.’s broader meta-analysis, the regional subgroup estimates were positive for Asia, the Americas, and Europe, although the European confidence interval included the null. Because that review combined total UPF measures with selected-food proxies, these estimates do not establish that the association differs by region. Comparisons are also limited by differences in populations, diets, exposure measures, and liver outcomes.

#### 3.6.5. How UPF Intake Was Reported

Most studies compared study-specific intake categories, such as tertiles, quartiles, or quintiles; others reported associations per increment in grams or percentage of energy or food weight. Thus, “low” and “high” intake did not represent the same amounts across studies. Henney et al. (2023) [28] reported little difference for moderate versus low UPF-related intake, but a stronger association for high versus low intake. That synthesis also included selected-food proxies. In the NOVA-restricted review by Zhang et al.(2025) [30], the highest versus lowest intake categories yielded a smaller pooled estimate. The available studies therefore support comparison of relative intake within each population, but do not establish a common low, moderate, or high UPF threshold for MASLD risk.

Taken together, the reviews identified a need for diverse prospective populations, consistent exposure and outcome definitions, and more complete adjustment for potential confounders, including social determinants. They also called for analysis of UPF subgroups and studies estimating the effects of replacing UPFs with minimally processed foods. These approaches could clarify which exposures are associated with MASLD and whether associations extend beyond steatosis to MASH, fibrosis, and disease progression.

### 3.7. Biological Plausibility of the Association Between UPF Consumption and MASLD

**Figure 2:**
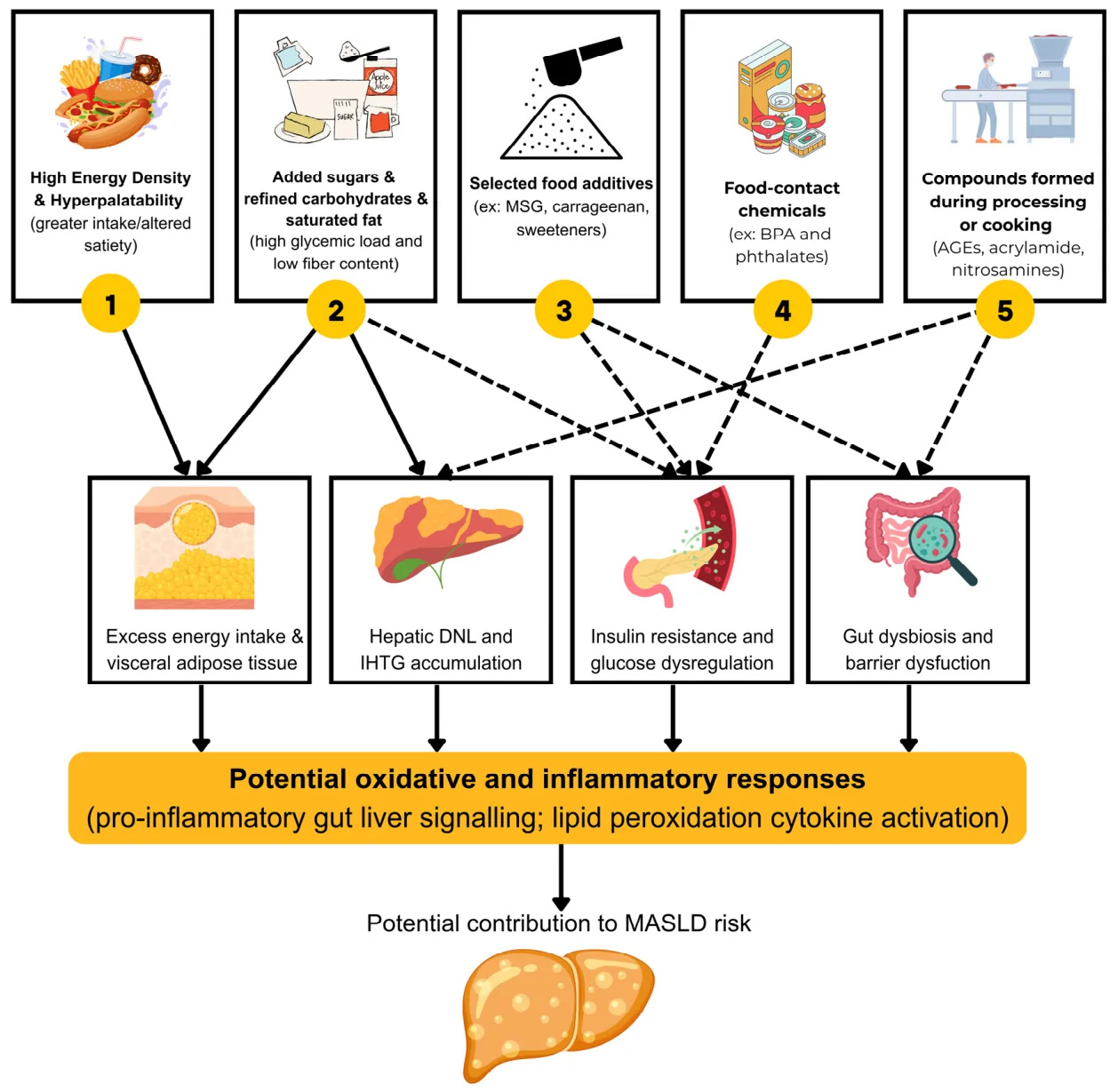
Proposed pathways linking selected characteristics of ultra-processed foods to MASLD. High energy intake and dietary composition may contribute to adiposity, hepatic lipid accumulation, and metabolic dysfunction. Additives, food-contact chemicals, and compounds formed during processing or cooking represent candidate exposures found in selected products. The dashed arrows denote pathways supported mainly by experimental or indirect evidence; their contribution to MASLD in humans remains to be established. Abbreviations: MSG, monosodium glutamate; BPA, bisphenol A; AGEs, advanced glycation end products; DNL, de novo lipogenesis; IHTG, intrahepatic triglycerides; MASLD, metabolic dysfunction-associated steatotic liver disease. Created with canva.com.

The available systematic reviews suggest that the association between UPF consumption and MASLD is biologically plausible through several interrelated nutritional and non-nutritional pathways (Figure 2). These pathways involve the nutritional composition and physical properties of some UPFs, as well as exposures associated with additives, packaging, and industrial processing. They may converge on excess hepatic lipid accumulation, insulin resistance, gut–liver signaling, oxidative stress, and inflammation. However, UPFs are heterogeneous, and evidence for a particular ingredient or contaminant should not be interpreted as evidence that all UPFs act through that mechanism.

#### 3.7.1. Excess Energy Intake and Visceral Adiposity

Many UPF products are formulated to be convenient and highly palatable, characteristics that may facilitate rapid eating and excess energy intake. In a controlled inpatient crossover trial, participants consumed approximately 500 kcal/day more during a UPF diet than during an unprocessed diet and gained weight over two weeks, despite the diets being matched for several presented nutrients [18]. Persistent positive energy balance could promote visceral adipose tissue expansion, increased delivery of fatty acids to the liver, and hepatic triglyceride accumulation. The trial establishes an effect on short-term energy intake and weight; it did not establish that the intervention caused MASLD.

#### 3.7.2. Added Sugars, Dietary Fat, and Hepatic Lipid Accumulation

Some UPF-rich diets contain substantial added sugars, refined carbohydrates, and saturated fat while displacing fiber-rich foods. Repeated exposure to these dietary patterns may favor insulin resistance and hepatic DNL. In the Framingham Heart Study, changes in sugar-sweetened beverage consumption were associated with subsequent MASLD, providing human longitudinal evidence relevant to this pathway [48]. Nevertheless, sugar-sweetened beverages are a specific UPF subgroup, so their association cannot be attributed to every product classified as UPF.

#### 3.7.3. Food Additives and Metabolic Regulation

Food additives and related compounds represent possible pathways beyond conventional nutrient composition, although evidence varies substantially by compound and experimental model. Carrageenan impaired insulin signaling through inflammatory mechanisms and increased expression of growth factor receptor-bound protein 10 (GRB10), an inhibitor of insulin receptor signaling [49]. Experiments involving non-caloric artificial sweeteners also reported changes in gut microbiota composition and glucose tolerance [50]. These findings concern metabolic outcomes and do not directly establish hepatic effects.

Animal experiments provide additional evidence concerning monosodium glutamate (MSG) and nitrosamines. Evidence suggests hepatic microsteatosis in MSG-exposed mice and altered hepatic inflammatory and lipid-related gene expression following combined MSG and trans-fat exposure [51]. However, exposure began during maternal feeding and continued in offspring, limiting extrapolation to habitual human consumption. Nitrosamines, which may form through reactions involving nitrite preservatives, are distinct from the preservatives themselves. It was reported that hepatic steatosis, inflammation and impaired insulin-related signaling occurred following *N*-nitrosodiethylamine exposure in rat pups [52]. The liver findings followed intraperitoneal injection rather than dietary exposure, further limiting their relevance to ordinary food consumption. Collectively, these findings support the proposed metabolic or hepatic pathways under specific experimental conditions. They do not establish that habitual dietary exposure to these compounds causes MASLD in humans or mediates the association between total UPF intake and liver disease.

#### 3.7.4. Food-Contact Chemicals

Packaging may introduce exposures that are not captured by a food’s nutrient profile. In a US population study, a greater dietary contribution from UPFs was associated with higher urinary concentrations of several phthalate metabolites and bisphenol F; the associations were not uniform across all measured compounds [53]. Separately, an in vitro and clinical study reported findings consistent with a possible role for bisphenol A in MASLD, particularly among people with T2DM [54]. Together, these studies suggest a potential route from food-contact materials to metabolic and hepatic effects, but they do not demonstrate that packaging chemicals mediate the observed UPF and MASLD association.

#### 3.7.5. Compounds Formed During Processing and Cooking

Heat treatment can generate advanced glycation end products (AGEs), while certain cooking processes can generate acrylamide. In a case–control study, higher estimated dietary AGE intake was associated with greater odds of MASLD [55]. In mice, a heat-processed, AGE-rich diet produced hepatic fat accumulation, oxidative stress, inflammation, and fibrosis, alongside impaired intestinal barrier function and altered gut microbial composition [56]. A US cross-sectional analysis also reported an association between acrylamide exposure and MASLD [57]. These studies provide different levels of evidence: the animal experiment examines a potential mechanism, whereas the human observational studies cannot establish whether these compounds caused liver disease.

#### 3.7.6. Gut–Liver Signaling and Downstream Inflammation

The intestinal microbiota and barrier provide a possible point of convergence for dietary composition, additives, and processing-derived compounds. Altered microbial activity or increased intestinal permeability could increase the delivery of inflammatory signals to the liver. The AGE-rich diet experiment provides direct animal evidence linking impaired gut homeostasis with hepatic lipid and inflammatory changes [56]; the sweetener experiments demonstrate that selected additives can alter microbiota-dependent glucose responses [50]. 

In a 90-day rat experiment, Shi et al. (2025) [58] reported hepatic lipid accumulation following UPF exposure, accompanied by changes in gut microbial composition and fecal metabolomic pathways. These findings provide experimental support for a potential gut–liver pathway linking UPF consumption to hepatic steatosis.

Processing-induced protein oxidation may represent an additional link between food composition and microbial responses. Food processing can generate amino acid oxidation products, including dityrosine, and alter protein structure and digestibility [59,60]. An in vitro fermentation study using human-derived gut microbiota further showed that thermally oxidized dough proteins altered microbial composition, including increases in potentially inflammation-associated taxa and decreases in potentially beneficial taxa [61]. This emerging evidence suggests a possible contribution of protein oxidation to gut–liver disturbances.

The biological relevance of these findings is supported by evidence linking gut–liver axis dysfunction to MASLD pathogenesis. Nutritional modulation of the gut–organ axis provides a broader context for these mechanisms. Dietary modulation of microbial metabolites, particularly short-chain fatty acids, may influence intestinal barrier integrity, endotoxin translocation, and inflammatory signaling involving nuclear factor kappa B (NF-κB)/mitogen-activated protein kinase (MAPK) and the NLR family pyrin domain-containing protein 3 (NLRP3) inflammasome. Although their review focuses on the gut–kidney axis, these pathways offer a complementary framework for considering diet-related gut–liver disturbances [62]. Lau et al. (2025) [63] describe intestinal barrier disruption, chronic inflammation, altered bile acid metabolism, and changes in microbial metabolites as potential contributors to disease development and progression. These mechanisms provide a framework for interpreting UPF-associated microbial and metabolomic alterations, although their specific contribution to the UPF–MASLD relationship remains to be established. The sequence from total NOVA-defined UPF intake to microbiome change, hepatic inflammation, and clinically confirmed MASLD has yet to be demonstrated in humans. Overall, the proposed mechanisms are biologically coherent but vary in evidential strength. Controlled human evidence supports an effect of an ultra-processed diet on energy intake, while experimental and observational studies support selected nutrient, additive, packaging, and processing-related pathways. Future studies should measure specific UPF subgroups and relevant exposures alongside repeated dietary assessments, metabolic and microbiome markers, and well-characterized MASLD outcomes. This would help determine which pathways account for associations observed with total UPF intake.

## 4. Discussion

This umbrella review aimed to critically synthesize evidence from systematic reviews and meta-analyses on the association between UPF consumption and MASLD in adult populations. Overall, the included evidence suggests that a higher consumption of UPFs is associated with a greater risk or prevalence of MASLD. However, this conclusion should be interpreted cautiously because the evidence base is largely observational, heterogeneous in exposure and outcome definitions, and partly overlapping across reviews. 

### 4.1. Evidence on UPF Consumption and MASLD

#### 4.1.1. Major Findings

The main finding of this umbrella review is that most quantitative syntheses reported a positive association between higher UPF intake and MASLD. Henney et al. (2023) [28] included nine studies involving 60,961 adults and found that a high UPF intake was associated with an increased MASLD risk. Zhang et al. (2025) [30], using a larger updated dataset of 10 articles with 513,440 participants and 20,637 MASLD cases, similarly reported that the highest UPF category was associated with a greater MASLD risk compared with the lowest category. Additionally, Zhang et al. (2025) [30] also reported a positive pooled estimate labeled “per 10% increment,” but the exposure units were not adequately harmonized in the published description and the separate formal dose–response model was not statistically significant. The categorical and continuous estimates therefore suggest an association without establishing a consistent exposure–response gradient.

Souza et al. (2026) [31] added a more temporally informative perspective by restricting their meta-analysis to prospective cohort studies and reporting that higher UPF consumption was associated with increased long-term MASLD risk. The consistency of these findings supports the interpretation that UPF intake is probably a relevant dietary marker for MASLD risk. However, the evidence is not strong enough to conclude causality. Grinshpan et al. (2024) [27] found that only three of six MASLD studies showed significant associations, although all large-scale prospective studies evaluating MASLD or metabolic syndrome showed positive findings. This pattern suggests that study design and statistical power are important. Positive findings appear more convincing in larger and prospective studies, whereas smaller and cross-sectional studies are more inconsistent.

#### 4.1.2. Consistency and Magnitude of Association

The direction of association was generally consistent, but the magnitude varied across reviews. Zhang et al. (2025) [30] reported a relatively modest association for highest versus lowest UPF intake, whereas Guo et al. (2025) [29] reported stronger pooled associations for adverse liver outcomes and MASLD. Differences in effect size are likely explained by differences in eligibility criteria, study design, exposure classification, outcome ascertainment, and included primary studies. Zhang et al. (2025) [30] specifically focused on NOVA-defined UPF exposure, whereas some earlier reviews included broader dietary exposures or UPF-related dietary patterns. This distinction matters because NOVA-defined UPF is conceptually different from fast food, processed meat, or a Western dietary pattern, although these exposures may overlap. Therefore, the appropriate conclusion is not that UPF has a single fixed-effect size for MASLD. Rather, the evidence indicates a positive association across multiple syntheses, with uncertainty around the exact magnitude of risk.

#### 4.1.3. Evidence by Study Design and Temporality

Temporality is one of the most important issues in this evidence base. Cross-sectional studies can show that UPF consumption and MASLD coexist, but they cannot determine whether UPF intake preceded liver fat accumulation. Reverse causation is possible because individuals with obesity, diabetes, or known fatty liver disease may change their diet after diagnosis. For this reason, prospective cohort studies are particularly important. Souza et al. (2026) [31] included only prospective cohort studies and found increased long-term MASLD risk with higher UPF intake. The primary studies cited in the updated literature also support this interpretation. Zhang et al. (2022) [33] reported that higher UPF consumption was associated with higher incident NAFLD risk in the Tianjin Chronic Low-grade Systemic Inflammation and Health cohort. Zhao et al. (2024) [21], using UK Biobank data, reported that higher UPF intake was associated with increased risk of NAFLD, liver fibrosis or cirrhosis, severe liver disease, and adverse liver biomarkers. These prospective findings provide stronger support than cross-sectional studies, although residual confounding remains possible [21,33].

The prospective evidence supports a temporal relationship, but it does not prove causality. UPF intake is strongly correlated with socioeconomic position, physical activity, total energy intake, obesity, metabolic syndrome, smoking, alcohol use, and overall diet quality. Even well-adjusted observational studies may not fully separate the effect of food processing from the broader behavioral and social pattern in which UPF intake occurs.

#### 4.1.4. Dose–Response Evidence

Dose–response evidence is important because it strengthens biological plausibility. Highest-versus-lowest intake comparisons and positive continuous associations do not, by themselves, establish a consistent dose–response relationship. Interpretation is limited by differences in exposure measurement and insufficient reporting of how units were harmonized. Grams per day, percentage of total energy intake, and percentage of total dietary weight represent distinct exposure measures and cannot be treated as interchangeable. Consequently, the available evidence does not establish the shape or magnitude of a common dose–response relationship or a precise harmful intake threshold. Further prospective studies using clearly defined exposure measures, repeated dietary assessments, and standardized liver outcomes are needed. Future dose–response syntheses should harmonize compatible exposure units transparently and evaluate energy-based and weight-based proportions separately.

#### 4.1.5. Advanced Liver Outcomes

The evidence is weaker and less consistent for advanced liver outcomes. Guo et al. (2025) [29] reported that UPF intake was associated with adverse liver outcomes overall, including MASLD, liver fibrosis, and liver cancer. However, that review also noted high heterogeneity and important limitations related to dietary assessment, outcome ascertainment, and UPF definitions. Souza et al. (2026) [31], which focused on prospective evidence, found no statistically significant association between higher UPF consumption and primary liver cancer, hepatocellular carcinoma, or intrahepatic cholangiocarcinoma. This distinction is important. The evidence appears more convincing for MASLD development than for liver cancer. Liver cancer is a less common outcome, requires longer follow-up, and is influenced by multiple intermediate pathways, including steatohepatitis, fibrosis, cirrhosis, viral hepatitis, alcohol exposure, diabetes, obesity, and genetic susceptibility. Therefore, the current evidence does not justify a strong conclusion that UPF independently increases liver cancer risk. A more careful interpretation is that UPF may contribute to metabolic and hepatic conditions that are upstream of severe liver disease, but direct evidence for cancer outcomes remains preliminary.

### 4.2. Synthesis of the Evidence and Biological Plausibility

Taken together, the five included reviews suggest an association between higher UPF consumption and adverse liver outcomes [27,28,29,30,31]. The proposed biological pathways make this association plausible, but they should be interpreted as complementary explanations rather than a single established mechanism. Greater energy intake, unfavorable dietary composition, additives, food-contact chemicals, and compounds formed during processing could influence hepatic lipid accumulation through overlapping metabolic and inflammatory processes. The contribution of each pathway is likely to differ across products and populations.

The strongest experimental evidence within this framework concerns energy intake. The controlled feeding trial by Hall et al. (2019) [18] showed that an UPF diet increased ad libitum intake and short-term weight gain. This supports a route through positive energy balance and adiposity, both of which are relevant to MASLD. Additional clinical context comes from a six-month randomized trial involving 173 adults with MASLD and overweight or obesity, of whom 148 completed follow-up [64]. Participants received a MedDiet, a low-carbohydrate, high-protein diet, or standard nutritional recommendations. Although changes in CAP did not differ significantly between randomized groups, reductions in BMI and NOVA-defined UPF intake were associated with CAP improvement in structural equation modeling. However, achieved UPF reduction was not randomized and may reflect overall dietary adherence or concurrent changes in energy intake and nutritional composition. These findings are therefore hypothesis-generating and do not establish a causal mediation pathway or an independent intervention effect of reducing UPF intake. Other studies provide evidence for more specific exposures: carrageenan impaired insulin signaling in experimental models [49], while an AGE-rich diet induced gut and liver abnormalities in mice [56]. Such findings identify mechanisms worth investigating, but they cannot establish that these exposures explain the associations between total UPF intake and MASLD in human cohorts. 

The distinction between an individual exposure and the overall UPF category is central to interpreting this literature. NOVA classifies foods according to the extent and purpose of processing; it does not indicate that every UPF has the same nutrient profile, additive combination, packaging, or cooking history. A mechanism demonstrated for sugar-sweetened beverages, carrageenan, or heat-generated AGEs may therefore apply to a subset of products rather than the entire category. Conversely, several pathways could act together within an individual’s habitual diet. Current observational studies generally cannot separate these possibilities because they measure total UPF consumption more often than the relevant chemical exposures or intermediate biological responses.

The apparent consistency across reviews also requires caution. The reviews share several primary studies, so their findings do not represent five independent bodies of evidence. Moreover, some included primary studies assessed processed meat, fast food, or other selected foods without measuring total NOVA-defined UPF intake. Differences in exposure definitions, dietary assessment, liver outcome ascertainment, and adjustment for energy intake and adiposity further limit comparisons. In particular, studies using historical NAFLD definitions or indirect liver-fat measures cannot automatically be interpreted as studies of clinically characterized MASLD or disease progression.

Future research should test the proposed pathways within the same participants rather than infer them from separate lines of evidence. Prospective studies could combine repeated, product-level NOVA assessments with measures of energy intake, UPF subgroups, packaging-related exposures, metabolic biomarkers, gut barrier function, and microbiome composition. Liver fat should be assessed with validated methods, while fibrosis and clinical outcomes should be examined separately. Future studies should explicitly distinguish the total effect of a defined change in UPF intake from associations conditional on adiposity, energy intake and dietary quality. Prespecified causal frameworks and temporally ordered measurements would help clarify the roles of these factors and whether UPF exposure provides information beyond measured nutritional composition. Persistence after adjustment alone should not be interpreted as evidence of a processing-specific causal effect. Until such studies are available, the biological mechanisms strengthen the plausibility of the observed association but do not establish which component of UPF consumption drives MASLD risk.

### 4.3. Methodological Limitations and Credibility of Evidence

Several limitations reduce certainty. First, dietary assessment was mostly self-reported. FFQs, 24-h recalls, and food records are prone to recall bias and measurement error. They also may not have been designed to classify foods by NOVA processing level. This can lead to exposure misclassification. Second, MASLD diagnosis varied across studies. Some studies used ultrasound, CAP, MRI, fatty liver indices, or hospital records. These methods do not identify identical disease states. For example, ultrasound may miss mild steatosis, while hospital records may capture more severe or clinically recognized disease. This variability likely contributes to heterogeneity. Third, residual confounding remains a major issue. UPF intake correlates with BMI, diabetes, physical inactivity, smoking, alcohol use, socioeconomic status, food insecurity, and overall dietary quality. Adjustment for BMI, total energy intake and dietary quality varied across the primary studies, complicating the comparison of their findings. Depending on the temporal and causal relationships, these variables may act as confounders, mediators, or both. Consequently, the most fully adjusted estimates do not necessarily address the same causal question and should not be interpreted as interchangeable estimates of the total effect of UPF intake. In this review, the reported estimates are summarized as study-specific adjusted associations rather than estimates of a common causal effect. Their persistence after adjustment does not establish an effect attributable specifically to food processing beyond nutritional composition. Because sequential adjustment models were not systematically compared, this synthesis cannot determine how additional adjustment changed the associations within each study. Future prospective studies should pre-specify the causal effect of interest, justify adjustment sets using explicit causal frameworks, and report sequential models to distinguish associations before and after adjustment for adiposity, energy intake and dietary quality. Fourth, overlap of primary studies across reviews limits the independence of the evidence. Review-level methodological quality should also be distinguished from certainty in the underlying associations. AMSTAR 2 assessed the methodology of the included systematic reviews rather than outcome-specific certainty. Souza et al. [31] rated the certainty of their pooled prospective evidence as very low, indicating substantial uncertainty around the estimated association despite its statistical significance. This rating pertains to their assessed evidence and should not be generalized automatically to all outcomes included here. No independent outcome-specific certainty assessment was undertaken in this umbrella review; findings were interpreted according to the study design, exposure definition, outcome ascertainment, and reported methodological limitations.

### 4.4. Interpretation in Relation to Broader UPF Evidence

The findings are consistent with the wider literature linking UPF exposure with cardiometabolic outcomes. Lane et al. (2024) [16], in a broad umbrella review of epidemiological meta-analyses, reported that greater UPF exposure was associated with a higher risk of several adverse health outcomes, particularly cardiometabolic, mental health, and mortality outcomes. However, they also noted that much of the evidence was low- or very low-quality using Grading of Recommendations Assessment, Development and Evaluation (GRADE). This broader evidence supports the plausibility of UPF as a risk marker for MASLD but also reinforces the need for cautious causal interpretation. The NOVA framework is useful for classifying foods by degree and purpose of industrial processing, but it is not without criticism. Monteiro et al. (2019) [13] define UPFs as industrial formulations involving substances derived from foods and additives, but classification can vary by dietary tool, country, and product formulation. This classification issue is relevant to this review because inconsistent UPF definitions across primary studies may affect comparability. 

### 4.5. Clinical and Public Health Implications and Future Research Priorities 

Reducing UPF intake may be reasonable as part of MASLD prevention and management, particularly when UPFs are replaced with minimally processed, nutrient-dense foods. However, the available evidence does not establish that UPF reduction alone prevents MASLD or identify a precise intake threshold. Practical substitutions include replacing sugar-sweetened beverages with water, packaged snacks and desserts with fruit or unsalted nuts, and frequent fast-food meals with meals based on vegetables, legumes, whole grains, and suitable protein sources. These changes align with MedDiet, DASH, and plant-forward dietary approaches while addressing overall dietary quality. Whether these substitutions improve liver outcomes beyond their effects on energy intake and body weight remains uncertain. The MedDiet pattern reduced hepatic steatosis and improved insulin sensitivity without weight loss [65]. This suggests that dietary composition may provide benefits beyond weight reduction, but the intervention did not isolate food-processing level. Similarly, differences were demonstrated in spontaneous energy intake and weight between ultra-processed and unprocessed diets, without establishing hepatic benefits independent of energy balance [18].

Clinically, UPF reduction should therefore complement individualized dietary management, including appropriate energy intake, weight management where indicated, and improved nutritional quality. Benefits will likely depend partly on which foods replace UPFs. A specific hepatic benefit attributable to reduced processing, independent of caloric restriction, weight change, and nutrient composition, requires confirmation in energy-matched, weight-maintaining trials with liver outcomes.

Future research should prioritize prospective cohort studies with repeated dietary assessment, standardized NOVA classification, validated MASLD outcomes, and prespecified causal frameworks that distinguish confounding from mediation. Studies should distinguish incident MASLD from disease severity and progression outcomes such as MASH, fibrosis, cirrhosis, and liver cancer. More work is also needed to determine whether specific UPF subtypes differ in risk. Sugar-sweetened beverages, processed meats, packaged snacks, ready meals, and industrial bakery products may not have equivalent associations with liver outcomes. Interventional studies would be particularly valuable. The field needs trials testing whether replacing UPFs with minimally processed foods reduces liver fat, liver enzymes, insulin resistance, inflammatory markers, or imaging-based MASLD outcomes. Such trials would help clarify whether UPF is a causal factor, a dietary marker, or both.

### 4.6. Limitation of the Umbrella Review

This umbrella review integrates review-level findings with primary-study mapping to clarify exposure definitions, liver outcomes, and methodological differences across the available evidence. Several considerations should guide interpretation. CCA quantified publication overlap, although participant overlap across separate reports was not formally assessed. AMSTAR 2 evaluated review-level methodology; no additional standardized primary-study risk-of-bias or outcome-specific certainty assessment was undertaken. Differences in dietary assessment, outcome ascertainment, and covariate adjustment limited direct comparison, and sequential adjustment models were not systematically examined. Mechanistic evidence provided complementary biological context rather than demonstrating mediation in humans. Within these boundaries, the review identifies recurring associations, distinguishes their evidential basis, and establishes priorities for more targeted prospective and intervention research.

## 5. Conclusions

In summary, the evidence synthesized in this umbrella review suggests that higher UPF consumption is associated with increased MASLD risk in adults. The association is supported by systematic reviews, updated meta-analyses, and prospective cohort studies. The evidence is more consistent for MASLD occurrence than for advanced liver outcomes such as liver fibrosis, cirrhosis, or liver cancer. Biological plausibility is supported by mechanisms involving excess energy intake, weight gain, visceral adiposity, insulin resistance, fructose-driven lipogenesis, gut microbiota disruption, inflammation, and oxidative stress. However, the evidence remains limited by observational design, exposure misclassification, heterogeneous outcome definitions, residual confounding, and overlap of primary studies. Therefore, UPF consumption should be interpreted as a plausible and modifiable dietary risk marker for MASLD, but current evidence is insufficient to establish causality or define a precise harmful threshold.

## Figures and Tables

**Table 1:** Characteristics and principal findings of systematic reviews evaluating ultra-processed food intake and MASLD.

Author (Year); Review Country	Publication and Search Date	MASLD Participants and Age	MASLD Papers and Study Design	Synthesis and Statistical Approach	Principal MASLD Findings	Conclusion Regarding Current Evidence
Henney et al., 2023; United Kingdom [28]	Published 10 May 2023; Medline and Web of Science searched through 5 December 2022	60,961 adults; ages across studies ranged approximately 18–90 years; all studies included men and women	9 studies: 3 cohort, 3 case–control, 3 cross-sectional. Only 4/9 assessed total UPF directly using NOVA; 5 used selected foods considered compatible with NOVA UPF, such as fast food, processed meat, or snacks	Random-effects meta-analysis using Cochran–Mantel–Haenszel; pooled fully adjusted RRs; Higgins’ *I*^2^ and Paule–Mandel τ^2^; fixed-effect meta-regression for dose response; subgroup/sensitivity analyses by design, NOVA use, sample size, region and dietary method; Begg funnel plots. Quality: Newcastle–Ottawa Scale and NutriGrade	Moderate vs. low intake: RR 1.03, 95% CI 1.00–1.07; *I*^2^ = 0%. High vs. low intake: RR 1.42, 95% CI 1.16–1.75; *I*^2^ = 89%. Seven of nine studies reported significant associations; the review interpreted the pattern as dose-responsive	The review supports a positive association between higher UPF-related intake and MASLD, but the estimate is weakened by high heterogeneity, inclusion of non-NOVA proxy exposures and limited longitudinal evidence. The evidence supports association, but not firm causality.
Guo et al., 2025; China [29]	Published 2025; PubMed, Cochrane Library, Embase and Web of Science searched through 17 October 2024	Approximately 426,863 study-level participant entries, calculated from the 14 MASLD rows. This is not a unique-person total because some cohorts overlap. Participants were mainly adults; the paper states ≥20 years, although its table includes an 18–90-year cohort and one mixed adolescent/adult sample	14 MASLD studies: 8 cohort, 3 case–control, 3 cross-sectional. Seven studies used NOVA; the remainder used processed meat, fast food, nutrient-poor snacks or dietary-pattern proxies	Adjusted ORs pooled using fixed effects when *p* > 0.10 and *I*^2^ ≤ 50%, or random effects when *I*^2^ > 50%; χ^2^ and *I*^2^ for heterogeneity; leave-one-out sensitivity analyses; subgroup analyses by continent and study design; funnel plots and Egger test; Stata 14. Quality: NOS for cohort/case–control and AHRQ for cross-sectional studies	Pooled MASLD association: OR 1.72, 95% CI 1.36–2.17; *I*^2^ = 93.3%. Subgroup estimates: Asia OR 1.47; America OR 1.99; Europe OR 1.89, although the European CI included the null. No individual study reversed the pooled estimate	The pooled estimate indicates higher MASLD odds, but it should not be interpreted as an estimate specifically for total NOVA UPF because half of the MASLD studies used selected foods or dietary patterns. The very high heterogeneity and mixture of cohort, case–control and cross-sectional evidence substantially limit causal and quantitative interpretation.
Zhang, Shu and Chen, 2025; China [30]	Published 11 July 2025; PubMed, Web of Science, Embase and CNKI searched through 31 March 2025	513,440 participants, including 20,637 NAFLD cases; adults approximately 18–90 years, although the review text generally describes participants as ≥20 years	10 observational studies: 6 cohort and 4 cross-sectional; all assessed total UPF using NOVA	HRs treated as RRs; ORs converted to RRs. Separate random-effects analyses of highest vs. lowest categories (10 papers, 11 estimates) and the source-labeled “each 10% increment” (6 papers, 7 estimates). Formal dose–response analysis: two-stage generalized least squares with restricted cubic splines at the 10th, 50th and 90th percentiles (6 studies reported, exact membership not enumerated with the curve). Subgroup, sensitivity and publication-bias analyses; Stata 17. Quality: NOS.	Highest vs. lowest UPF: RR 1.22, 95% CI 1.14–1.31; *I*^2^ = 78.5%. Each 10% increment: RR 1.06, 95% CI 1.04–1.09; *I*^2^ = 75.9%. The formal dose–response model was not statistically significant: RR 1.02, 95% CI 0.98–1.07; *p* = 0.295. Estimates were positive in both cohort and cross-sectional subgroups	This synthesis restricted exposure to NOVA-defined total UPF and found a positive highest versus lowest association with NAFLD. Its positive estimate labeled “per 10% increment” has an insufficiently specified common exposure unit. Despite the authors’ description of a linear trend, the formal model was not statistically significant. A consistent dose–response relationship, causality and a precise intake threshold remain unestablished
Souza et al., 2026; Brazil [31]	Accepted 13 November 2025 and published in a 2026 issue; PubMed and Embase searched through 5 September 2025	377,772 participants in four MASLD cohorts; mean age 54.4 years, 57.0% women	4 prospective cohort papers, all using NOVA. Three were pooled for the highest-vs-lowest comparison; one severe-MASLD study was summarized separately	Random-effects model with restricted maximum likelihood and Hartung–Knapp adjustment; pooled multivariable-adjusted HRs; *I*^2^; leave-one-out analyses and Baujat plots; R 4.4.0. Only prospective cohorts with >1-year follow-up and time-to-event data were eligible. Quality: ROBINS-I; certainty: GRADE	Highest vs. lowest UPF, based on three cohorts: HR 1.32, 95% CI 1.11–1.58; *I*^2^ = 50.3%. Severe MASLD: HR 1.26, 95% CI 1.11–1.43. Continuous analyses were consistently positive: 4% higher risk per 100 g/day, 6% per SD in the Chinese cohort, and 9–10% per SD in the Korean cohort. GRADE certainty was very low	This provides the clearest evidence of temporality: higher NOVA-defined UPF intake precedes incident MASLD. The estimated long-term increase is moderate, around 32% for the highest versus lowest intake. Nevertheless, only three studies contributed to the main pooled result and certainty remains very low, so causality and intake thresholds are not established.
Grinshpan et al., 2024; Israel [27]	Available online 17 November 2023; published in JHEP Reports 2024; search completed March 2023	29,675 enrolled participants, calculated across the six MASLD/liver-fat papers; ages approximately 18–90 years. Only 13 of the 20 Hall-trial participants underwent liver MRS	6 papers: 1 crossover RCT, 2 prospective analyses and 3 cross-sectional studies	No quantitative pooling. Narrative synthesis was selected because of variation in UPF assessment, liver outcomes and the small number of studies. Studies were tabulated by outcome and design. Quality: JBI tools for observational studies and RoB 2 for the RCT	3/6 studies reported a significant positive association. Both large prospective studies were positive. Two small cross-sectional studies were null after adjustment. The 13-person RCT liver-fat subsample showed no significant change after a 14-day UPF diet. Associations in positive studies remained after accounting for BMI and energy intake	Evidence suggested that total NOVA-defined UPF may be a MASLD risk factor, but the review considered the evidence insufficient for a firm conclusion. Prospective evidence was more consistent than cross-sectional evidence, while interventional evidence was essentially absent.

Legend: AHRQ, Agency for Healthcare Research and Quality; CI, confidence interval; CNKI, China National Knowledge Infrastructure; GRADE, Grading of Recommendations Assessment, Development and Evaluation; HR, hazard ratio; *I*^2^, I-squared heterogeneity statistic; JBI, Joanna Briggs Institute; MASLD, metabolic dysfunction-associated steatotic liver disease; MRS, magnetic resonance spectroscopy; NOS, Newcastle–Ottawa Scale; NOVA, food classification according to the extent and purpose of processing; OR, odds ratio; RCT, randomized controlled trial; ROBINS-I, Risk Of Bias In Non-randomized Studies—of Interventions; RR, relative risk; SD, standard deviation; UPF, ultra-processed food. Participant and paper counts refer only to the MASLD or liver-fat evidence considered in each review. For Guo et al., the participant figure represents study-level entries and may include overlapping cohorts. Grinshpan et al. used narrative synthesis without meta-analysis.

**Table 2:** Primary Research Papers Included in Each Systematic Review.

Primary Research Paper Author (Year)	Henney et al. (2023)	Guo et al. (2025)	Zhang et al. (2025)	Souza et al. (2026)	Grinshpan et al. (2024)
Ivancovsky-Wajcman et al. (2021) [32]	✓	✓	✓	—	✓
2. Zhang S. et al. (2022) [33]	✓	✓	✓	✓	✓
3. Fridén et al. (2022) [34]	✓	—	✓	—	✓
4. Konieczna et al. (2022) [35]	✓	✓	✓	—	✓
5. Liu et al. (2023) [36]	—	✓	✓	—	✓
6. Zhao, Martinez Steele et al. (2023) [37]	—	✓	✓	—	—
7. Zhang Y.F. et al. (2024) [38]	—	✓	✓	✓	—
8. Zhao Clay-Gilmour et al. (2024) [21]	—	✓	✓	✓	—
9. Canhada et al. (2024) [39]	—	—	✓	—	—
10. Fu et al. (2025) [40]	—	—	✓	✓	—
11. Hall et al., 2019 [18]	—	—	—	—	✓
12. Zelber-Sagi et al. (2018) [41]	✓	✓	—	—	—
13. Noureddin et al. (2020) [42]	✓	✓	—	—	—
14. Yari et al. (2020) [43]	✓	✓	—	—	—
15. Tutunchi et al. (2021) [44]	—	✓	—	—	—
16. Odegaard et al. (2022) [45]	✓	✓	—	—	—
17. Rahimi-Sakak et al. (2022) [46]	✓	✓	—	—	—
18. Xiao et al. (2023) [47]	—	✓	—	—	—

Table legend. ✓, primary paper included in the corresponding review; —, primary paper not included. Primary papers are identified by author and publication year only. Counts refer only to primary papers contributing to MASLD or liver-fat outcomes.

**Table 3:** Primary publication overlap across systematic reviews, overall and stratified by exposure definition.

Evidence Group	Distinct Primary Publications (*r*)	Total Publication Appearances (*N*)	Reviews Included (*c*)	CCA (%)	Degree of Overlap
All included primary publications	18	43	5	**34.72**	Very high
Strictly NOVA-defined UPF exposures	11	31	5	**45.45**	Very high
Proxy exposures—all five reviews	7	12	5	**17.86**	Very high
Proxy exposures—restricted to contributing reviews ^1^	7	12	2	**71.43**	Very high

Legend: CCA, corrected covered area; NOVA, food-processing classification system; UPF, ultra-processed food. CCA was calculated as [(N − r)/(r × c − r)] × 100, where *N* represents the total number of publication appearances across reviews, including repeated appearances; *r*, the number of distinct primary publications; and *c*, the number of reviews included in the calculation. Values above 15% indicate very high overlap (Pieper et al., 2014) [22]. Calculations are based on the corrected inclusion matrix. ^1^ Only Henney et al. (2023) and Guo et al. (2025) contributed proxy-exposure publications.

**Table 4:** Characteristics of studies assessing total ultra-processed food intake using the NOVA classification.

Study; Country	Design, Follow-Up and Population	Dietary Assessment and Validation	UPF Exposure	Adjustment/Experimental Comparison
**Ivancovsky-Wajcman et al. (2021) [32]** **Israel**	Cross-sectional screening study. Hepatic screening study among volunteers undergoing colonoscopy/health screening. Follow-up: Not applicable. n = 789; 52.6% men; mean age 58.83 ± 6.58 years; 305 with ultrasound-defined NAFLD	Tool: 117-item semiquantitative food-frequency questionnaire General validation: The questionnaire was adapted to the Israeli population; explicit validation details for the exact FFQ were not fully reported in the paper Processing/NOVA validation: No. NOVA classification was applied retrospectively to FFQ items; the tool was not designed or validated specifically for processing-level assessment	Total NOVA Group 4 intake as percentage of total energy; high intake mainly defined above the median (~28% energy), with additional categorical analyses	Age, sex, BMI, saturated-fat and protein intake, physical activity, coffee intake, and fiber intake
**Zhang S. et al. (2022) [33]** **China**	Prospective cohort. TCLSIH Cohort. Follow-up: Median 4.2 years. n = 16,168 adults; both sexes; age 18–90 years	Tool: Validated semiquantitative FFQ: 81 items initially and expanded to 100 items; previous-month intake General validation: Yes. Reproducibility and validity were examined against a repeat FFQ and four 4-day weighed dietary records Processing/NOVA validation: No. The FFQ was validated for energy, nutrients and food groups, not specifically for processing degree; NOVA was assigned to listed items post hoc.	Total NOVA Group 4 intake; primary metric energy-adjusted g/1000 kcal/day; also g/day, percentage energy and percentage weight	Age, sex, BMI, smoking, alcohol, education, occupation, household income, physical activity, family history, depressive symptoms, energy intake, healthy-diet score, hypertension, diabetes and hyperlipidemia
Fridén et al. (2022) [34] Sweden	Cross-sectional population-based study. POEM cohort; cross-sectional imaging analysis. Follow-up: Not applicable. n = 286; men and women; all approximately 50 years old	Tool: 140-item semiquantitative FFQ assessing habitual intake over the previous year General validation: Partial. A shorter version had been validated against weighed dietary records and fatty-acid biomarkers Processing/NOVA validation: No. It was not designed or validated to determine processing level; NOVA assignment was retrospective	NOVA Group 4 intake expressed as percentage of total energy and analyzed in tertiles	Sex, education, physical activity, smoking, protein, alcohol, saturated fat, polyunsaturated fat, fiber, total sugar and BMI
Konieczna et al. (2022) [35] Spain	Prospective observational analysis within a randomized lifestyle trial. PREDIMED-Plus multicenter cohort. Follow-up: 1 year; diet and liver indices repeatedly assessed at baseline, 6 and 12 months. n = 5867; men and women aged 55–75 years; all had overweight/obesity and metabolic syndrome	Tool: Validated 143-item semiquantitative FFQ administered face-to-face by trained dietitians General validation: Yes. The FFQ had been validated for habitual dietary intake in a Spanish population Processing/NOVA validation: No. The authors explicitly noted that the FFQ was not designed to capture food-processing details	Total NOVA Group 4 intake, mainly percentage of total food weight (g/day), analyzed as concurrent change	Age, sex, intervention group, follow-up time, education, smoking, height, repeatedly measured physical activity, sedentary behavior and alcohol; sensitivity models included energy and dietary factors
Liu et al. (2023) [36] United States	Cross-sectional nationally representative survey. NHANES 2011–2018. Follow-up: Not applicable. n = 6545 adults; 3045 men and 3500 women; mean age 49.33 years	Tool: Up to two interviewer-administered 24-h dietary recalls using USDA Food and Nutrient Database coding; 89.7% completed two recalls General validation: Standardized national dietary method; the paper did not present a separate validation specifically for this analysis Processing/NOVA validation: No. The authors explicitly stated that the interviews were not designed for NOVA and processing information was not systematically collected	NOVA Group 4 as percentage of total food and beverage weight; quartiles and each 10% increment	Primary confounder model (model 2): age, sex, race/ethnicity, education, income-to-poverty ratio, marital status, smoking, BMI, ALT, fasting triglycerides, total cholesterol and uric acid. Separate models additionally considered dietary mediators.
**Zhao, Martinez Steele et al. (2023) [37]** **United States**	Cross-sectional nationally representative survey. NHANES 2017–2018. Follow-up: Not applicable. Adult analysis: n = 2734, both sexes, age ≥ 20 years; 35.6% had NAFLD. The paper also included 806 adolescents.	Tool: Two 24-h dietary recalls collected using standardized NHANES procedures General validation: Standardized dietary recall method; no processing-specific validation reported Processing/NOVA validation: No. Food-processing classification was applied retrospectively to recall foods	Total NOVA Group 4 intake, mainly energy-adjusted g/day and quintiles; percentage energy analyses were also performed	Age, sex, race/ethnicity, education, smoking pack-years, alcohol, physical activity and energy intake; BMI and waist circumference were evaluated as mediators
Zhang Y.-F. et al. (2024) [38] United Kingdom	Prospective cohort. UK Biobank. Follow-up: Median 10.5 years. n = 143,073; both sexes; age 40–69 years at recruitment	Tool: Web-based self-administered Oxford WebQ/24-h dietary recall, with repeated invitations; average based on at least two recalls General validation: Yes for general food and nutrient estimation: reliability was comparable with interviewer-administered 24-h recall Processing/NOVA validation: No. The tool was not created as a processing-specific instrument; uncertain foods required assumptions during NOVA coding	Total NOVA-defined UPF in grams/day, averaged from at least two 24-h recalls; quartiles and continuous analyses. The primary measure was absolute intake, rather than percentage dietary weight.	Age, sex, ethnicity, Townsend deprivation, education, BMI, smoking, alcohol-consumption frequency and physical activity
**Zhao, Clay-Gilmour et al. (2024) [21]** **United Kingdom**	Prospective cohort. UK Biobank. Follow-up: Median 8.9 years. n = 173,889; men and women aged 40–69 years; biomarker analysis n = 137,173	Tool: Modified web-based 24-h dietary recalls covering 206 foods and 32 beverages, repeated up to five times General validation: General reproducibility and dietary validity were supported by prior UK Biobank studies Processing/NOVA validation: No. The recall was not specifically designed to classify processing level; NOVA was assigned retrospectively	Total NOVA Group 4 intake using all available recalls; g/day, energy-adjusted intake and quartiles; UPF subgroups also examined	Age, sex, ethnicity, deprivation, smoking, alcohol, physical activity, BMI, aspirin use, diabetes and energy intake
Canhada et al. (2024) [39] Brazil	Prospective ELSA-Brasil occupational cohort. Baseline cohort n = 15,105, aged 35–74 years; MASLD analysis n = 8699, with 1893 incident cases. Mean follow-up for MASLD: 7.9 years (SD 1.3).	Validated 114-item baseline FFQ covering the previous year. General dietary validation reported; the FFQ was not designed specifically for NOVA classification.	Overall NOVA-defined UPF and prespecified subgroups, expressed in g/day; associations per 1-SD increase (271 g/day for MASLD)	Age, sex, race/color, education, per-capita income, smoking, leisure-time physical activity and alcohol; BMI and hypertension were not included in the principal model because they may mediate the association; energy-adjusted sensitivity analysis
Fu et al. (2025) [40] South Korea	Prospective cohort. Health Examinees (HEXA), Korean Genome and Epidemiology Study. Follow-up: Median 4.2 years. n = 44,642; 12,075 men and 32,567 women; age 40–69 years; 1562 incident cases	Tool: Validated 106-item semiquantitative FFQ General validation: Yes. Validated against 12-day dietary records; median correlation approximately 0.39, with additional seasonal records for reproducibility Processing/NOVA validation: No. The validation concerned usual dietary intake, not food-processing degree; NOVA was assigned after data collection	Total NOVA Group 4 intake and six UPF subgroups; primary sex-specific quartiles, with g/day and alternative energy/weight metrics in sensitivity analyses	Age, energy intake, other NOVA groups, income, education, physical activity, alcohol, smoking, sleep, stress and baseline obesity, diabetes, hypertension and dyslipidemia
Hall et al. (2019) [18] United States	Randomized inpatient crossover feeding trial; n = 20 weight-stable adults (10 men, 10 women), mean age 31.2 years (SE 1.6). Two 14-day diet periods; liver-fat measurements available in 13 participants.	All foods provided; intake determined by weighing foods offered and leftovers. No self-reported dietary instrument.	NOVA-defined ultra-processed versus unprocessed menus, consumed ad libitum. Menus were designed to match presented calories, macronutrients, sugar, sodium and fiber.	Within-person diet comparison. Liver fat was a secondary measurement; the primary endpoint was energy intake.

Legend: General dietary validation concerns estimation of foods or nutrients and does not establish validity for processing-level classification. Abbreviations: ALT, alanine aminotransferase; BMI, body mass index; ELSA-Brasil, Brazilian Longitudinal Study of Adult Health; FFQ, food-frequency questionnaire; HEXA, Health Examinees; MASLD, metabolic dysfunction-associated steatotic liver disease; NAFLD, non-alcoholic fatty liver disease; NHANES, National Health and Nutrition Examination Survey; POEM, Prospective Investigation of Obesity, Energy and Metabolism; PREDIMED-Plus, Prevención con Dieta Mediterránea–Plus; SD, standard deviation; SE, standard error; TCLSIH, Tianjin Chronic Low-grade Systemic Inflammation and Health; UK, United Kingdom; UPF, ultra-processed food; USDA, United States Department of Agriculture; WebQ, web-based dietary questionnaire. NOVA is the name of the food-processing classification system, not an acronym. Units and symbols: g, grams; kcal, kilocalories; n, number of participants.

**Table 5:** Liver-outcome definitions and their correspondence with current MASLD criteria in NOVA-defined ultra-processed food studies.

Study; Country	Original Outcome/Endpoint	Steatosis or Liver-Outcome Assessment	Cardiometabolic Assessment and Diagnostic Role	Alcohol Assessment and Other Exclusions	Correspondence with Current MASLD Criteria
**Ivancovsky-Wajcman et al. (2021) [32]** **Israel**	NAFLD; prevalent ultrasound-defined steatosis. Presumed NASH and fibrosis markers are separate secondary outcomes.	Abdominal ultrasound using standardized criteria. FibroMax: quantitative NashTest 2 ≥0.5 for presumed moderate/severe NASH; FibroTest ≥0.31 for at least borderline-significant fibrosis (≥F1–F2, as described by authors). No biopsy.	Fasting glucose/lipids, BP, waist and diabetes were assessed; metabolic syndrome was defined separately. A cardiometabolic factor was not required for NAFLD case status.	Structured face-to-face questionnaire; excluded ≥30 g/day in men or ≥20 g/day in women, HBV/HCV and suspected drug-induced steatosis.	Partial correspondence: imaging and alcohol exclusions are compatible components; ≥1 current cardiometabolic criterion was not required. Presumed NASH is not histologically confirmed MASH.
Zhang S. et al. (2022) [33] China	Incident NAFLD.	Ultrasound at follow-up every 1–2 years; ≥2 of bright liver, deep signal attenuation and vascular blurring.	Measured BMI/waist, BP, fasting glucose and lipids; diabetes, hypertension and hyperlipidaemia considered as covariates. No metabolic entry criterion for NAFLD.	Alcohol/lifestyle questionnaire; excluded ≥210 g/week in men or ≥140 g/week in women. Baseline liver diseases excluded.	Partial correspondence; steatosis and alcohol components assessed, but current metabolic criteria were not required. No retrospective MASLD confirmation.
Fridén et al. (2022) [34] Sweden	Continuous MRI liver fat; authors also reported an operational NAFLD prevalence.	1.5-T MRI quantified liver fat. The secondary NAFLD label was based solely on hepatic fat >5.5%; competing causes were not fully established.	BMI and other characteristics measured; no cardiometabolic requirement in the liver-fat outcome or secondary threshold definition.	Alcohol obtained from 140-item FFQ and adjusted as percentage energy. Competing-cause data were incomplete; one man with liver fat >5.5% consumed >30 g/day.	Liver-fat phenotype only; the >5.5% threshold alone does not establish NAFLD or MASLD etiology. The authors’ disease label is therefore qualified by incomplete etiologic assessment.
Konieczna et al. (2022) [35] Spain	Repeated FLI and HSI values (NAFLD-related surrogate indices).	FLI uses BMI, waist, triglycerides and GGT; HSI uses ALT/AST, BMI, diabetes and sex. Continuous change at baseline, 6 and 12 months; not imaging-confirmed steatosis.	All participants had overweight/obesity and ≥3 metabolic-syndrome components. Repeated anthropometry, BP and fasting metabolic tests were obtained.	FFQ-derived g/day, repeatedly measured; excluded alcohol abuse/addiction defined as >50 g/day during the preceding 6 months.	Metabolically enriched population, but no direct steatosis confirmation. Alcohol exclusion is less restrictive than MASLD-specific sex thresholds and may permit MetALD-range consumption.
Liu et al. (2023) [36] United States	Prevalent NAFLD, operationalized using US-FLI.	US-FLI ≥30; an algorithm-defined probable-steatosis endpoint, not imaging or histology in this analysis.	NHANES anthropometric and laboratory data, including BMI, glucose, insulin and lipids, were available; no separate ≥1 cardiometabolic criterion for case status.	Excluded >21 drinks/week in men or >14 drinks/week in women and HBV/HCV. Drink-based thresholds are not directly equivalent to sex-specific g/day thresholds.	Incomplete correspondence: surrogate steatosis and no required current metabolic criterion; drink-based alcohol thresholds are not a direct MASLD reclassification.
**Zhao, Martinez Steele et al. (2023) [37]** **United States**	Prevalent NAFLD; NAFLD with/without clinically significant fibrosis (CSF), assessed separately.	VCTE CAP ≥285 dB/m for steatosis; LSM ≥8.6 kPa for CSF. Adult and adolescent analyses were separate. No histological staging.	Measured BMI/waist and self-reported physician-diagnosed diabetes; metabolic factors were covariates/mediators, not an entry requirement.	Questionnaire on alcohol use; excluded >3 standard drinks/day in men or >2 in women, HBV/HCV and steatogenic medications.	Partial correspondence; CAP-defined steatosis but no metabolic requirement. Drink thresholds differ from explicit MASLD g/day limits. LSM-defined CSF is not biopsy stage or observed progression.
Zhang Y.-F. et al. 2024 [38] (2024) United Kingdom	Authors’ term: “severe NAFLD.” Operational endpoint: NAFLD/NASH-related hospitalization or death.	Linked hospital/death records; ICD-10 K76.0 and K75.8. No standardized baseline/follow-up imaging, biopsy or fibrosis-stage requirement for this endpoint.	BMI measured and adjusted; no cardiometabolic case criterion reported.	Baseline alcohol-consumption frequency adjusted; a quantitative sex-specific alcohol exclusion is not stated in the supplied main report. Baseline liver disease excluded.	MASLD correspondence unconfirmed. The record-based “severe NAFLD” endpoint does not demonstrate advanced fibrosis or progression from known steatosis.
**Zhao, Clay-Gilmour et al. (2024) [21]** **United Kingdom**	Record-defined NAFLD; separately, fibrosis/cirrhosis, liver cancer and severe liver disease.	NAFLD: ICD-10 K75.8/K76.0; fibrosis/cirrhosis: K74.0–K74.6. Severe liver disease is a broader composite, including alcoholic liver diagnoses; it is not MASLD-specific.	BMI and self-reported diabetes adjusted; baseline glycaemic/lipid biomarkers measured in a subset. No required cardiometabolic case criterion.	Questionnaire alcohol frequency adjusted (never/occasional, 1–2, 3–4, ≥5 times/week). No quantitative alcohol exclusion reported in the main methods; baseline hepatitis/cirrhosis excluded.	MASLD correspondence unconfirmed: codes do not establish steatosis plus metabolic and alcohol criteria. The severe composite is not MASLD-specific or measured fibrosis progression.
Canhada et al. (2024) [39] Brazil	Incident MASLD, as defined by the authors.	FLI > 60 plus ≥ 1 of five cardiometabolic factors; index-defined probable steatosis, not imaging.	BMI ≥ 25 (≥23 for Asians) or waist ≥ 94 cm men/>80 cm women; dysglycaemia/T2D; BP ≥ 130/85 or treatment; TG ≥ 150 mg/dL or treatment; HDL ≤ 40 men/≤50 women mg/dL or treatment. Standardized anthropometry/BP, fasting glucose/lipids, OGTT, HbA1c and medication/history data.	Structured alcohol interview; g/week included in adjustment. A sex-specific alcohol exclusion is not specified in the supplied outcome definition.	Explicit metabolic/steatosis operationalization using MASLD terminology. Full correspondence remains uncertain because the report does not specify a MASLD alcohol restriction; full equivalence is not established.
Fu et al. (2025) [40] South Korea	Incident NAFLD (FLI-defined).	FLI cut-off 60, operationalized as FLI ≥ 60; baseline fatty liver excluded.	BMI/waist, fasting lipids/glucose, BP and disease/treatment history assessed. Obesity, diabetes, hypertension and dyslipidaemia were covariates; no ≥1 metabolic case criterion.	Self-administered questionnaire: never/former versus current drinking; history of alcohol abuse excluded. Quantitative abuse threshold not specified in the supplied main text.	Incomplete correspondence: NAFLD terminology and surrogate endpoint; neither a required current metabolic criterion nor quantitative MASLD alcohol restriction was established.
Hall et al. (2019) [18] United States	Liver-fat content/change; no NAFLD, MAFLD or MASLD case definition.	T1/T2-corrected proton MRS on a 3-T scanner; baseline and end of each 14-day period (n = 13).	Anthropometry and metabolic tests measured; diabetes excluded. No metabolic-risk or steatosis requirement for enrollment.	Inpatient feeding study; no quantitative alcohol eligibility threshold specified in the supplied article’s methods.	Experimental liver-fat evidence only. Not a trial of MASLD occurrence, treatment or fibrosis progression.

Legend: Original disease terminology and operational definitions are retained as reported in each study. Correspondence with current MASLD criteria represents a qualitative assessment of the reported steatosis, cardiometabolic and alcohol-related components; no participant-level reclassification was performed. Measurement of, or adjustment for, cardiometabolic factors does not establish that these factors were required for diagnosis. Imaging-derived liver fat, index-defined probable steatosis, administrative diagnoses and non-invasive fibrosis markers are distinguished. “Severe NAFLD” denotes the original study’s hospitalization/death endpoint and does not imply formally staged fibrosis or demonstrated disease progression. “Not specified” indicates that information was not stated in the supplied report, rather than confirming that the procedure was not performed. Abbreviations: ALD, alcohol-related liver disease; ALT, alanine aminotransferase; AST, aspartate aminotransferase; BMI, body mass index; BP, blood pressure; CAP, controlled attenuation parameter; CSF, clinically significant fibrosis; FLI, Fatty Liver Index; GGT, gamma-glutamyl transferase; HbA1c, glycated hemoglobin; HBV, hepatitis B virus; HCV, hepatitis C virus; HDL, high-density lipoprotein; HSI, Hepatic Steatosis Index; ICD-10, International Classification of Diseases, 10th Revision; LSM, liver stiffness measurement; MAFLD, metabolic dysfunction-associated fatty liver disease; MASLD, metabolic dysfunction-associated steatotic liver disease; MASH, metabolic dysfunction-associated steatohepatitis; MetALD, metabolic dysfunction-associated steatotic liver disease with increased alcohol intake; MRI, magnetic resonance imaging; MRS, magnetic resonance spectroscopy; NAFLD, non-alcoholic fatty liver disease; NASH, non-alcoholic steatohepatitis; NHANES, National Health and Nutrition Examination Survey; OGTT, oral glucose tolerance test; T2D, type 2 diabetes; TG, triglycerides; UPF, ultra-processed food; US-FLI, United States Fatty Liver Index; VCTE, vibration-controlled transient elastography. NOVA is the name of the food-processing classification system, not an acronym.

**Table 6:** Liver-outcome findings and methodological appraisal of NOVA-defined ultra-processed food studies, stratified by outcome ascertainment.

Study	Original Disease Terminology and Operational Outcome	Corresponding Liver Findings	Strengths	Limitations and Terminology Appraisal
A. Imaging/CAP-defined NAFLD				
Ivancovsky-Wajcman et al. (2021) [32]	NAFLD; ultrasound-defined prevalence. Presumed NASH and fibrosis assessed separately by FibroMax, without biopsy.	No significant association with whole-sample prevalent ultrasound-defined NAFLD. Within NAFLD, high UPF intake was associated with presumed NASH (OR 1.89, 95% CI 1.07–3.38). Among ever-smokers with NAFLD, the OR for FibroTest-defined fibrosis was 2.85 (1.14–7.14). These are cross-sectional biomarker associations.	Large screening sample; standardized ultrasound; detailed metabolic phenotyping; evaluation of NASH/fibrosis markers and smoking interaction	Cross-sectional design; retrospective NOVA coding; residual confounding. Ultrasound-defined NAFLD did not require a current metabolic-risk criterion. Biomarker-defined NASH/fibrosis must not be equated with histology.
Zhang S. et al. (2022) [33]	NAFLD; incident ultrasound-defined disease.	3752 incident cases. Highest versus lowest UPF quartile: HR 1.18 (95% CI 1.07–1.30); per 1-SD increase of 62.7 g/1000 kcal/day: HR 1.06 (1.03–1.09).	Prospective design; large sample; validated FFQ; standardized ultrasound; broad confounder adjustment; several sensitivity analyses	Single baseline dietary assessment; FFQ not processing-specific; potential NOVA misclassification; ultrasound cannot stage NASH/fibrosis; residual confounding; one Chinese urban cohort Interpretation: Core eligible prospective study for total NOVA-defined UPF and incident NAFLD
Zhao, Martinez Steele et al. (2023) [37]	NAFLD; CAP ≥285 dB/m. Clinically significant fibrosis defined separately by LSM ≥8.6 kPa.	Adults: Q5 versus Q1 NAFLD OR 1.72 (95% CI 1.01–2.93). An ordinal analysis of non-NAFLD, NAFLD without CSF and NAFLD with CSF yielded OR 1.74 (1.06–2.85); not a longitudinal progression estimate.	National sample; objective transient elastography; two 24-h dietary recalls; mediation analysis; liver and inflammatory biomarkers	Cross-sectional; retrospective NOVA coding; no required metabolic factor; drink-based alcohol exclusions. Adult n = 2734 and adolescent n = 806 findings must remain separate. CAP/LSM are non-invasive measurements. Exploratory cross-sectional mediation analyses cannot establish temporal ordering or causal pathways through adiposity.
B. Quantitative liver-fat outcomes; no confirmed MASLD diagnosis				
Fridén et al. (2022) [34]	Quantitative MRI liver fat. Authors’ secondary NAFLD prevalence used liver fat >5.5% alone, without full etiologic assessment.	Fully adjusted association with log-transformed liver fat: β 0.005 (95% CI −0.01 to 0.02), *p* = 0.47. The positive visceral-fat association is a different outcome.	Objective MRI measurement; population-based sample; exposure expressed as percentage energy; detailed body-fat phenotyping.	Small cross-sectional sample; incomplete competing-cause assessment; no MASLD metabolic case criteria. Imaging liver fat is not automatically etiologically classified liver disease.
Hall et al. (2019) [18]	MRS liver-fat content/change; no NAFLD, MAFLD, MASLD or NASH case definition.	MRS subsample n = 13: liver fat 1.2% (SE 0.1) at baseline, 0.95% (0.1) after the unprocessed diet and 1.1% (0.2) after the UPF diet. Neither end-period value differed significantly from baseline (*p* = 0.24 and 0.74, respectively).	Randomized crossover feeding; objectively measured intake; proton MRS.	Small MRS subsample; low baseline liver fat; 14 days per diet; no MASLD diagnosis; trial powered for energy intake. No evidence on fibrosis progression.
C. Index-defined NAFLD, MASLD and changes in steatosis indices				
Konieczna et al. (2022) [35]	NAFLD-related FLI/HSI values; continuous surrogate-index changes rather than disease onset.	Each 10-percentage-point increase in UPF dietary weight share over one year was associated with +1.60 FLI points (95% CI 1.24–1.96) and +0.43 HSI points (0.29–0.57). The authors reported substantial proportions mediated by BMI/waist changes; these exploratory, model-based estimates involve anthropometric score components and concurrent changes and do not establish causal mediation of independently measured liver fat.	Prospective repeated exposure and index measurements; large multicenter sample; validated FFQ; component, sensitivity and stratified analyses.	FLI contains BMI and waist circumference; HSI contains BMI, but not waist circumference. Mediation models involving BMI with either score, or waist with FLI, are susceptible to mathematical coupling. Waist-HSI analyses lack this direct shared term but remain closely related to BMI. Findings concern composite scores, not independently measured liver-fat change or established causal mediation.
Liu et al. (2023) [36]	NAFLD; US-FLI ≥30 (index-defined probable steatosis).	Primary confounder model (model 2): Q4 versus Q1 OR 1.83 (95% CI 1.33–2.53); per 10-percentage-point higher dietary UPF weight share OR 1.15 (1.09–1.22). These cross-sectional analyses do not establish temporal ordering or causal mediation.	Large nationally representative sample; survey weighting; two recalls for most participants; mediation and sensitivity analyses	US-FLI contains waist circumference, age, race/ethnicity, GGT, fasting insulin and glucose; BMI is not a component. Associations may reflect adiposity/metabolic inputs rather than independently measured liver fat. Cross-sectional design, residual confounding and retrospective NOVA coding remain limitations.
Canhada et al. (2024) [39]	MASLD; author-defined FLI >60 plus ≥1 cardiometabolic risk factor. Alcohol restriction not specified in the reported definition.	Per 271 g/day (1 SD) higher total UPF intake: MASLD RR 1.12 (95% CI 1.08–1.16). Per subgroup-specific SD: baked/fried snacks RR 1.05 (1.01–1.09), processed meat 1.09 (1.05–1.13), sweetened beverages 1.09 (1.05–1.12).	Prospective diverse cohort; standardized metabolic measurements; validated dietary tool; explicit metabolic component of the MASLD outcome; subgroup analyses.	FLI incorporates BMI, waist circumference, triglycerides and GGT; the separate metabolic criterion may also use related measurements. This endpoint is not independent imaging evidence of steatosis. FFQ/NOVA misclassification, residual confounding, and unstated MASLD-specific alcohol restriction remain limitations.
Fu et al. (2025) [40]	NAFLD; incident FLI-defined probable steatosis (cut-off 60).	Q4 versus Q1: NAFLD HR 1.35 (95% CI 1.06–1.71) in men and 1.48 (1.19–1.86) in women; 1562 incident FLI-defined cases.	Large prospective Asian cohort; sex-stratified analyses; validated FFQ; numerous sensitivity metrics; examination of individual UPF categories	FLI incorporates BMI and waist circumference, so incident threshold crossing may partly reflect adiposity changes rather than independently measured steatosis. Single baseline FFQ, residual confounding and unstated quantitative alcohol abuse threshold remain limitations. Source ramen subgroup estimates are internally inconsistent.
**D. Administrative-record NAFLD diagnosis**				
**Zhao, Clay-Gilmour et al. (2024) [21]**	NAFLD; administrative-record K75.8/K76.0 diagnoses.	1108 record-defined NAFLD cases. Q4 versus Q1 HR 1.43 (95% CI 1.21–1.70); approximately 4% higher risk per 100 g/day.	Very large prospective cohort; repeated recalls; long follow-up; multiple liver outcomes and objective biomarkers; extensive sensitivity analyses	Administrative under-ascertainment; no explicit quantitative alcohol restriction; residual confounding; potential UK Biobank sample overlap. Do not interpret case detection as measured fibrosis progression.
**E. NAFLD/NASH-related hospitalization or death**				
**Zhang Y.-F. et al. 2024 [38]**	“Severe NAFLD”; authors’ term for NAFLD/NASH-related hospitalization or death identified by K76.0/K75.8.	Authors’ “severe NAFLD” endpoint: 1445 hospital/death-record cases. Q4 versus Q1 HR 1.26 (95% CI 1.11–1.43).	Very large prospective cohort; long follow-up; repeated dietary recalls; linked clinical outcomes; subgroup and sensitivity analyses	Record ascertainment may miss undiagnosed/milder disease. K76.0/K75.8 do not require advanced fibrosis. No serial staging or progression from established NAFLD was measured. Potential population overlap with the other UK Biobank report.

Legend: Original disease terminology is retained, and findings are grouped by outcome ascertainment. Estimates are from the principal adjusted models unless otherwise stated. Adjustment sets differed across studies; the estimates therefore represent study-specific adjusted associations and should not be interpreted as estimates of a common total or direct causal effect. Adjustment for adiposity, energy intake, dietary quality or nutrients does not by itself isolate an effect attributable to processing. Liver-fat measurements, steatosis indices, non-invasive fibrosis markers and record-based diagnoses are not interchangeable. “Severe NAFLD” denotes hospitalization or death, not formally staged fibrosis or demonstrated progression. Abbreviations: BMI, body mass index; CAP, controlled attenuation parameter; CI, confidence interval; CSF, clinically significant fibrosis; FFQ, food-frequency questionnaire; FLI, Fatty Liver Index; HR, hazard ratio; HSI, Hepatic Steatosis Index; ICD-10, International Classification of Diseases, 10th Revision; LSM, liver stiffness measurement; MAFLD, metabolic dysfunction-associated fatty liver disease; MASLD, metabolic dysfunction-associated steatotic liver disease; MASH, metabolic dysfunction-associated steatohepatitis; MRI, magnetic resonance imaging; MRS, magnetic resonance spectroscopy; NAFL, non-alcoholic fatty liver; NAFLD, non-alcoholic fatty liver disease; NASH, non-alcoholic steatohepatitis; OR, odds ratio; Q, quantile; RR, risk ratio; SD, standard deviation; SE, standard error; UK, United Kingdom; UPF, ultra-processed food; US-FLI, United States Fatty Liver Index; β, regression coefficient. NOVA is a classification-system name, not an acronym.

**Table 7:** Study characteristics and liver-related findings for proxy dietary exposures.

Study; Country	Design and Population	Exposure and Dietary Method	Original Outcome Findings
**Zelber-Sagi et al. (2018); Israel [41]**	Cross-sectional Tel Aviv screening study; n = 789, aged 40–70 years; mean age 58.83 years (SD 6.58), 52.6% men. Detailed meat questionnaire: n = 357.	Red and processed meat intake, cooking methods and heterocyclic amines. Tool: 117-item FFQ plus a detailed meat-consumption and cooking-method questionnaire.	High red and/or processed meat consumption was associated with NAFLD: OR 1.47 (95% CI 1.04–2.09).
Noureddin et al. (2020); United States [42]	Nested case–control study within the prospective Multiethnic Cohort. Medicare-linked NAFLD: 2974 cases (518 with cirrhosis), 29,474 matched controls. Median interval from baseline diet to first NAFLD claim: 17.4 years.	Red meat, processed red meat, poultry, processed poultry, cholesterol and fiber, generally expressed per 1000 kcal. Tool: Validated quantitative FFQ.	Highest versus lowest processed red-meat quartile: OR 1.18 (95% CI 1.05–1.32); total red meat OR 1.15, with generally stronger associations for cirrhosis.
Yari et al. (2020); Iran [43]	Case–control study in a tertiary hepatology clinic; 143 newly diagnosed NAFLD cases and 471 ultrasound-negative controls (n = 614).	Energy-dense nutrient-poor snacks: cakes/biscuits, sweets/chocolate, salty snacks and soft drinks; percentage of energy and quartiles. Tool: Validated 168-item semiquantitative FFQ assessing the previous year.	Total snacks Q4 versus Q1: adjusted NAFLD OR 2.27 (95% CI 1.19–4.31). Cakes/biscuits and soft drinks had positive trend tests, but their extreme-quartile CIs included 1; those individual extreme-quartile contrasts were not significant.
Tutunchi et al. (2021); Iran [44]	Age- and sex-matched case–control study, Imam Reza Hospital; 105 newly diagnosed NAFLD cases and 105 controls, aged 30–60 years (n = 210).	Factor-derived VLFD and SHMS patterns from a three-day food diary (two weekdays and one weekend day), analyzed with Nutritionist IV. Patterns analyzed in tertiles; this was not a 24-h recall or total NOVA exposure.	SHMS highest versus lowest tertile: age-stratified adjusted ORs 2.93 (95% CI 1.54–5.12), 3.17 (1.04–6.66), and 4.22 (1.22–6.91), for ages 30–39, 40–49 and 50–59 years, respectively. VLFD was inversely associated with NAFLD.
Odegaard et al. (2022); United States [45]	Prospective CARDIA cohort; repeated fast-food assessment over 25 years. CT adipose analysis n = 3156; liver attenuation/MAFLD analysis n = 3001, including 302 MAFLD cases.	Average frequency of meals from named fast-food restaurants, from never/≤1 per month to ≥3 per week. Tool: Repeated single fast-food-frequency question at years 0, 7, 10, 15, 20 and 25; separate validated CARDIA Diet History at selected examinations.	Fast food ≥3 times/week versus never/≤1 time/month: MAFLD OR 5.18 (95% CI 2.87–9.37). Higher intake was also associated with lower CT liver attenuation (greater liver fat). MAFLD was measured at year 25, not established as incident MASLD.
**Rahimi-Sakak et al. (2022); Iran [46]**	Clinic-based case–control study in Tehran; 196 NAFLD cases and 803 controls (n = 999), matched within 5-year age ranges.	Red meat and processed meat intake in quartiles. Tool: Reliable and validated semiquantitative FFQ.	Highest versus lowest quartile: red meat OR 3.65 (95% CI 1.85–7.18); processed meat OR 3.25 (1.57–6.73).
Xiao et al. (2023); China [47]	Cross-sectional baseline analysis of the Gongcheng longevity cohort; n = 2664 adults >30 years (1016 men, 1648 women); 241 NAFLD cases.	Traditional Chinese, Western, and cereal-potato dietary patterns Tool: Face-to-face semiquantitative FFQ; dietary patterns derived by factor analysis.	Model 1 Q4 versus Q1: Western pattern NAFLD OR 2.799 (95% CI 1.620–4.837); cereal-potato pattern OR 0.581 (0.371–0.910). These are not the additionally BMI-adjusted model 2 estimates.

Legend: Original disease terminology is retained. Estimates are adjusted unless otherwise specified; proxy exposures and differing liver outcomes should not be interpreted as equivalent. Abbreviations: BMI, body mass index; CARDIA, Coronary Artery Risk Development in Young Adults; CI, confidence interval; CT, computed tomography; FFQ, food-frequency questionnaire; MAFLD, metabolic dysfunction-associated fatty liver disease; NAFLD, non-alcoholic fatty liver disease; OR, odds ratio; Q, quartile; SD, standard deviation; SHMS, sweets–hydrogenated fats–red and processed meat–soft drinks dietary pattern; UPF, ultra-processed food; VLFD, vegetable–legume–fruit–low-fat dairy dietary pattern. NOVA is a classification-system name, not an acronym.

**Table 8:** Liver-outcome definitions and their correspondence with current MASLD criteria in proxy-exposure studies.

Study	Original Outcome/Endpoint	Steatosis/Liver-Outcome Ascertainment	Cardiometabolic Assessment	Alcohol Assessment and Exclusions	Correspondence With Current MASLD Criteria
Zelber-Sagi et al. (2018); [41]	Prevalent NAFLD; imaging-defined.	Standardized abdominal ultrasound by one radiologist; hepatorenal index measured. No required metabolic criterion.	Fasting glucose/HbA1c, insulin/HOMA-IR, BMI and other metabolic characteristics assessed; diabetes defined by tests/medication.	Structured interview; excluded ≥30 g/day men or ≥20 g/day women, viral markers and suspected drug-induced steatosis.	Partial correspondence; ≥1 current metabolic criterion was not required. Potentially shared Tel Aviv screening population with Ivancovsky-Wajcman; not independent confirmation.
Noureddin et al. (2020) [42]	Claims-defined NAFLD, with/without claims-defined cirrhosis.	Medicare chronic liver disease: one inpatient or ≥2 outpatient/carrier claims on different dates. NAFLD assigned after excluding coded other etiologies, with BMI ≥30, diabetes or NAFLD codes. Cirrhosis/complication codes analyzed separately.	BMI and physician-diagnosed diabetes from baseline questionnaire. Obesity/diabetes were one route to attribution, not required for every coded NAFLD case.	Baseline questionnaire; >21 drinks/week men or >14 women reclassified as alcoholic liver disease; coded alternative liver etiologies excluded.	Incomplete correspondence: claims-based attribution, no uniform imaging or ≥1 current metabolic criterion; preserve drinks/week. Cirrhosis is claims-defined, not serial fibrosis staging.
Yari et al. (2020) [43]	NAFLD case–control status; ultrasound plus FibroScan.	Ultrasound-positive cases referred for FibroScan. Authors report CAP >263 and “fibrosis score >7” as diagnostic criteria; units for the latter are not stated in the report. Controls had no ultrasound steatosis.	BMI measured and included in adjustment; no required metabolic criterion. Other clinical history collected by interview.	Alcohol included in adjustment. <20 g/day men and <10 g/day women; detailed quantitative instrument not specified in this brief report.	Partial correspondence; steatosis/alcohol components reported but no required current metabolic factor. The reported fibrosis score does not establish a histological stage.
Tutunchi et al. (2021) [44]	NAFLD case–control status; imaging-defined moderate/severe steatosis.	One expert radiologist; ultrasound echogenicity grading. Moderate/severe hepatic steatosis, not moderate/severe fibrosis.	Measured BMI/waist, BP-related history, fasting glucose/lipids and insulin; diabetes and hypertension were exclusion criteria, not diagnostic components.	Any alcohol intake in the preceding 6 months excluded; history obtained in person. Other liver diseases and relevant drugs excluded.	Partial correspondence: no alcohol exposure in stated window, but no required current metabolic criterion. Ultrasound steatosis grade does not establish fibrosis stage.
Odegaard et al. (2022) [45]	MAFLD at year 25; CT attenuation also analyzed continuously.	CT mean liver attenuation ≤40 HU plus overweight/obesity, T2D, or normal weight with ≥2 metabolic abnormalities. CT threshold indicates at least moderate/severe steatosis.	Standardized anthropometry/BP and glucose/lipid tests; medication/history. Lean criterion included waist, BP, TG, HDL, prediabetes, HOMA-IR ≥2.5 or hsCRP >2 mg/L.	Repeated beverage-specific questions; intake calculated as mL ethanol/day. Excluded >60 mL/day at year 25, relevant drugs, hepatitis/cirrhosis history and HIV.	MAFLD is not equivalent to MASLD: metabolic algorithms differ and >60 mL/day alcohol exclusion permits intake above MASLD limits. MASLD reclassification was not performed.
Rahimi-Sakak et al. (2022) [46]	NAFLD case–control status; ultrasound/CAP.	Gastroenterologist diagnosis, ultrasound and FibroScan CAP >263 for cases; controls had no ultrasound steatosis. No biopsy-based stage.	Measured BMI; diabetes and other medical history by questionnaire. Metabolic factors were adjustment variables, not required for diagnosis.	Questionnaire; cases required <30 g/day men or <20 g/day women. Control criteria specify no ultrasound steatosis; identical alcohol criterion is not explicitly restated for controls.	Partial correspondence for cases, but no required current metabolic criterion. A fully reclassified MASLD population was not established.
Xiao et al. (2023) [47]	NAFLD according to the operational Methods definition.	Moderate/severe steatosis on ultrasound; no recent steatogenic drugs, hepatotoxin exposure or bariatric surgery. No formal fibrosis assessment.	Measured BMI/waist, BP, fasting glucose, HbA1c and lipids; hypertension/diabetes characterized but not required for case status.	Trained interviewers used a lifestyle questionnaire; excluded >20 g/day men or >10 g/day women and history of viral hepatitis.	Partial correspondence; no current metabolic entry criterion. The isolated MAFLD wording in the Discussion is not supported by an operational MAFLD definition; retain Methods-defined NAFLD.

Legend: Original disease terminology and diagnostic definitions are retained. Correspondence with MASLD criteria is assessed qualitatively; no participant-level reclassification was performed. Steatosis severity, fibrosis and cirrhosis are distinguished. “Not specified” indicates information not reported in the supplied article. Abbreviations: BMI, body mass index; BP, blood pressure; CAP, controlled attenuation parameter; CT, computed tomography; HbA1c, glycated hemoglobin; HDL, high-density lipoprotein; HOMA-IR, homeostasis model assessment of insulin resistance; hsCRP, high-sensitivity *C*-reactive protein; HU, Hounsfield units; ICD, International Classification of Diseases; IR, insulin resistance; MAFLD, metabolic dysfunction-associated fatty liver disease; MASLD, metabolic dysfunction-associated steatotic liver disease; NAFLD, non-alcoholic fatty liver disease; T2D, type 2 diabetes; TG, triglycerides.

**Table 9:** Methodological limitations of the MASLD evidence base and priorities for future research.

Systematic Review	Methodological Limitations Arising from the Included MASLD Studies	Priority Future Directions
Henney et al., 2023 [28]	Only nine studies and three longitudinal studies; the longitudinal-only result lost statistical significance, probably because of low power. Five studies did not assess total NOVA UPF but used selected foods as proxies. High heterogeneity in design, exposure definition, dietary tool and MASLD diagnosis. Most studies used FFQs, creating recall and under-reporting bias. Ultrasound/FibroScan had limited sensitivity and could not adequately stage disease. UPF was expressed using incompatible units and study-specific quantiles, preventing a universal threshold. Cross-sectional and case–control evidence was vulnerable to reverse causality and selection bias.	Larger prospective cohorts; repeated diet assessments; standardized NOVA coding; harmonized exposure metrics such as percentage of energy and grams/1000 kcal; more sensitive liver assessment with MRI-PDFF, elastography and validated fibrosis measures; assessment of disease severity; analyses by UPF subgroup and substitution with minimally processed foods.
Guo et al., 2025 [29]	Extreme heterogeneity for MASLD, *I*^2^ = 93.3%. Only seven studies used NOVA; others assessed processed meat, fast food, snacks or broad dietary patterns, making the pooled exposure conceptually inconsistent. Mixed cohort, case–control and cross-sectional designs were pooled as ORs. Diagnostic methods ranged from ultrasound and CAP to FLI and ICD records. Possible overlapping cohorts were not clearly resolved in the reported MASLD total. Adjustment sets differed substantially. The review did not establish a standardized MASLD-specific participant total or exposure threshold	Restrict future syntheses to total NOVA Group 4 exposure or analyze non-NOVA proxies separately; prospectively register strict eligibility rules; avoid double counting overlapping cohorts; use longitudinal designs, standardized NOVA definitions and harmonized exposure units; employ sensitive imaging and comprehensive diagnostic methods; identify which UPF subgroups or components are most relevant.
Zhang et al., 2025 [30]	All studies were observational. Four were cross-sectional. FFQs and 24-h recalls were not designed or validated specifically for NOVA classification. Exposure categories and cut-off points differed. Outcome ascertainment varied among FLI, ultrasound, CAP and hospital records. Residual confounding remained possible. Gray literature was not searched extensively. Heterogeneity remained high after subgroup and sensitivity analyses. Eight of ten studies came from developed countries, limiting generalizability.	Large prospective cohorts in diverse low-, middle- and high-income populations; repeated, processing-specific dietary assessment; standardized UPF metrics; incident MASLD outcomes rather than only prevalent or surrogate outcomes; harmonized confounder models; validated imaging or clinical outcomes; intervention or substitution studies where feasible; better examination of non-linear dose–response relationships.
Souza et al., 2026 [31]	Only four MASLD cohorts and only three in the main pooled estimate. Dietary intake was generally measured once, which may not represent long-term exposure. FFQs and recalls remain vulnerable to measurement error. Differences in populations, cultural diets, exposure units and quantile definitions generated heterogeneity. Residual confounding, including social determinants, could not be excluded. Most outcomes used FLI or ICD codes rather than histology or direct quantitative imaging. The small number of studies prevented subgroup analysis, meta-regression, UPF-category analysis and a harmonized dose–response model. Certainty was rated very low.	Large, diverse prospective cohorts; repeated technology-based dietary assessments; product-level NOVA coding; harmonized absolute and relative UPF units; direct imaging-based incident MASLD outcomes; investigation of individual UPF subgroups; effect modification by genetics, environmental exposures, metabolic status and social determinants; studies estimating replacement effects when UPF is substituted with minimally processed foods.
Grinshpan et al., 2024 [27]	Few MASLD studies, with half cross-sectional; reverse causality and residual confounding were therefore major concerns. All dietary methods (observational studies) were self-reported, and none was specifically validated to capture food processing or NOVA categories. The only RCT was very small, short and not designed primarily for MASLD. No study used liver biopsy; diagnostic methods varied. Very few studies assessed MASH or fibrosis. Publication bias could not be excluded. The heterogeneity and small evidence base precluded quantitative pooling.	Prospective and interventional studies; dietary instruments specifically designed for food-processing assessment; product-level web or mobile dietary tools; validated clinical liver outcomes; repeated diet measurement; mechanistic studies addressing additives, packaging chemicals, glycemic response, inflammation, oxidative stress and the microbiome; assessment of MASH, fibrosis and progression rather than steatosis alone.

Legend: Limitations refer to the primary MASLD studies included in each review, not to the methodological quality of the review itself. Abbreviations: CAP, controlled attenuation parameter; FFQ, food-frequency questionnaire; FLI, Fatty Liver Index; ICD, International Classification of Diseases; MASLD, metabolic dysfunction-associated steatotic liver disease; MASH, metabolic dysfunction-associated steatohepatitis; MRI-PDFF, magnetic resonance imaging proton density fat fraction; OR, odds ratio; RCT, randomized controlled trial; UPF, ultra-processed food; *I*^2^, percentage of variability across study estimates attributable to heterogeneity rather than sampling error.

## Data Availability

No new data were created or analyzed in this study. Data sharing is not applicable to this article.

## Supplementary Materials

The following supporting information can be downloaded at: https://www.mdpi.com/article/10.3390/nu18213585/s1, Supplementary Table S1. Methodological quality of systematic reviews on ultra-processed food intake and MASLD assessed using AMSTAR 2.

## Abbreviations

AGEs, advanced glycation end products; AHRQ, Agency for Healthcare Research and Quality; ALD, alcohol-related liver disease; ALT, alanine aminotransferase; AMSTAR 2, A Measurement Tool to Assess Systematic Reviews 2; AST, aspartate aminotransferase; BMI, body mass index; BP, blood pressure; BPA, bisphenol A; CAP, controlled attenuation parameter; CARDIA, Coronary Artery Risk Development in Young Adults; CCA, corrected covered area; CI, confidence interval; CNKI, China National Knowledge Infrastructure; CSF, clinically significant fibrosis; CT, computed tomography; DASH, Dietary Approaches to Stop Hypertension; DNL, de novo lipogenesis; DOI, digital object identifier; ELSA-Brasil, Brazilian Longitudinal Study of Adult Health; FFQ, food-frequency questionnaire; FLI, Fatty Liver Index; GGT, gamma-glutamyl transferase; GRADE, Grading of Recommendations Assessment, Development and Evaluation; GRB10, growth factor receptor-bound protein 10; HbA1c, glycated hemoglobin; HBV, hepatitis B virus; HCV, hepatitis C virus; HDL, high-density lipoprotein; HEXA, Health Examinees; HIV, human immunodeficiency virus; HOMA-IR, homeostasis model assessment of insulin resistance; HR, hazard ratio; hsCRP, high-sensitivity *C*-reactive protein; HSI, Hepatic Steatosis Index; HU, Hounsfield units; ICD, International Classification of Diseases; ICD-10, International Classification of Diseases, 10th Revision; IHTG, intrahepatic triglycerides; IR, insulin resistance; JBI, Joanna Briggs Institute; LSM, liver stiffness measurement; MAFLD, metabolic dysfunction-associated fatty liver disease; MAPK, mitogen-activated protein kinase; MASH, metabolic dysfunction-associated steatohepatitis; MASLD, metabolic dysfunction-associated steatotic liver disease; MedDiet, Mediterranean diet; MetALD, metabolic dysfunction-associated steatotic liver disease with increased alcohol intake; MRI, magnetic resonance imaging; MRI-PDFF, magnetic resonance imaging proton density fat fraction; MRS, magnetic resonance spectroscopy; MSG, monosodium glutamate; NAFL, non-alcoholic fatty liver; NAFLD, non-alcoholic fatty liver disease; NASH, non-alcoholic steatohepatitis; NF-κB, nuclear factor kappa B; NHANES, National Health and Nutrition Examination Survey; NLRP3, NLR family pyrin domain-containing protein 3; NOS, Newcastle–Ottawa Scale; OGTT, oral glucose tolerance test; OR, odds ratio; PMID, PubMed identifier; POEM, Prospective Investigation of Obesity, Energy and Metabolism; PREDIMED-Plus, Prevención con Dieta Mediterránea–Plus; PRISMA, Preferred Reporting Items for Systematic Reviews and Meta-Analyses; Q, quantile or quartile, as specified; RCT, randomized controlled trial; ROBINS-I, Risk Of Bias In Non-randomized Studies of Interventions; RR, risk ratio; SD, standard deviation; SE, standard error; SHMS, sweets–hydrogenated fats–red and processed meat–soft drinks dietary pattern; T2D, type 2 diabetes; T2DM, type 2 diabetes mellitus; TCLSIH, Tianjin Chronic Low-grade Systemic Inflammation and Health; TG, triglycerides; UK, United Kingdom; UPF, ultra-processed food; UPFs, ultra-processed foods; US, United States; USDA, United States Department of Agriculture; US-FLI, United States Fatty Liver Index; VCTE, vibration-controlled transient elastography; VLFD, vegetable–legume–fruit–low-fat dairy dietary pattern; WebQ, web-based dietary questionnaire.
