# Supplementary material for: Ultra-Processed Food Consumption and Metabolic Dysfunction-Associated Steatotic Liver Disease (MASLD): Evidence Synthesis, Methodological Appraisal, and Mechanistic Insights
Source: Nutrients. 2026 Oct 29;18(21):3585. doi: 10.3390/nu18213585 (PMC13764997; doi:10.3390/nu18213585)
Supplement: Supplementary file 1 [file nutrients-18-03585-s001.zip › nutrients-4640153-supplementary.pdf]

Supplementary Table S1. Methodological quality of systematic reviews on ultra-processed food intake and MASLD assessed using AMSTAR 2

AMSTAR 2 item	Henney et al. (2023)	Guo et al. (2025)	Zhang et al. (2025)	Souza et al. (2026)	Grinshpan et al. (2024)
1. Did the research questions and eligibility criteria include the components of PECO?	Y	N	Y	Y	Y
2. Was the review protocol established before conducting the review, with deviations justified? †	Partial Y	Partial Y	N	N	Partial Y
3. Did the authors explain their selection of eligible study designs?	N	N	N	Y	N
4. Did the authors use a comprehensive literature-search strategy? †	Partial Y	Partial Y	Partial Y	Partial Y	Partial Y
5. Was study selection performed in duplicate?	Y	Y	Y	Y	Y
6. Was data extraction performed in duplicate?	Y	Y	Y	N	N
7. Did the authors provide a list of excluded full-text studies and justify each exclusion? †	N	N	N	Partial Y	Y
8. Were the included studies described in adequate detail?	Y	Partial Y	Partial Y	Y	Y
9. Was a satisfactory method used to assess risk of bias in individual studies? †	Partial Y	Partial Y	Partial Y	Y	Partial Y
10. Did the authors report the funding sources of the included primary studies?	N	N	N	N	N
11. If meta-analysis was performed, were appropriate statistical methods used? †	Y	N	Y	Y	NA
12. Did the authors assess the potential effect of study-level risk of bias on the pooled results?	N	N	Y	N	NA
13. Did the authors account for risk of bias when interpreting the results? †	Y	N	Y	Y	Y
14. Did the authors provide a satisfactory explanation of heterogeneity?	Y	Y	Y	Y	Y
15. If quantitative synthesis was performed, was publication bias investigated and discussed? †	Y	Y	Y	N	NA
16. Did the authors report potential conflicts of interest and funding for the review?	Y	N	Y	Y	Y
Overall Rating	**Low**	**Critically low**	**Critically low**	**Critically low**	**Moderate**

Legend. AMSTAR 2, A Measurement Tool to Assess Systematic Reviews 2; PECO, population, exposure, comparator, and outcome; Y, yes; Partial Y, partial yes; N, no; NA, not applicable. †Critical AMSTAR 2 domain.
